# Eps8 Regulates Axonal Filopodia in Hippocampal Neurons in Response to Brain-Derived Neurotrophic Factor (BDNF)

**DOI:** 10.1371/journal.pbio.1000138

**Published:** 2009-06-30

**Authors:** Elisabetta Menna, Andrea Disanza, Cinzia Cagnoli, Ursula Schenk, Giuliana Gelsomino, Emanuela Frittoli, Maud Hertzog, Nina Offenhauser, Corinna Sawallisch, Hans-Jürgen Kreienkamp, Frank B. Gertler, Pier Paolo Di Fiore, Giorgio Scita, Michela Matteoli

**Affiliations:** 1 Department of Medical Pharmacology, National Research Council (CNR) Institute of Neuroscience, University of Milan, Milan, Italy; 2 Filarete Foundation, Milan, Italy; 3 IFOM Foundation – FIRC (Italian Foundation for Cancer Research) Institute of Molecular Oncology, Milan, Itlay; 4 Institut für Humangenetik, Universitätsklinikum Hamburg-Eppendorf, Hamburg, Germany; 5 Massachusetts Institute of Technology, Koch Institute, Cambridge, Massachusetts, United States of America; 6 Department of Experimental Oncology, Istituto Europeo di Oncologia, Milan, Italy; 7 University of Milan Medical School, Milan, Italy; 8 Institute for Hospitalisation and Scientific Care (IRCCS) Don Gnocchi, Milan, Italy; Cambridge University, United Kingdom

## Abstract

The regulation of filopodia plays a crucial role during neuronal development and synaptogenesis. Axonal filopodia, which are known to originate presynaptic specializations, are regulated in response to neurotrophic factors. The structural components of filopodia are actin filaments, whose dynamics and organization are controlled by ensembles of actin-binding proteins. How neurotrophic factors regulate these latter proteins remains, however, poorly defined. Here, using a combination of mouse genetic, biochemical, and cell biological assays, we show that genetic removal of Eps8, an actin-binding and regulatory protein enriched in the growth cones and developing processes of neurons, significantly augments the number and density of vasodilator-stimulated phosphoprotein (VASP)-dependent axonal filopodia. The reintroduction of Eps8 wild type (WT), but not an Eps8 capping-defective mutant, into primary hippocampal neurons restored axonal filopodia to WT levels. We further show that the actin barbed-end capping activity of Eps8 is inhibited by brain-derived neurotrophic factor (BDNF) treatment through MAPK-dependent phosphorylation of Eps8 residues S624 and T628. Additionally, an Eps8 mutant, impaired in the MAPK target sites (S624A/T628A), displays increased association to actin-rich structures, is resistant to BDNF-mediated release from microfilaments, and inhibits BDNF-induced filopodia. The opposite is observed for a phosphomimetic Eps8 (S624E/T628E) mutant. Thus, collectively, our data identify Eps8 as a critical capping protein in the regulation of axonal filopodia and delineate a molecular pathway by which BDNF, through MAPK-dependent phosphorylation of Eps8, stimulates axonal filopodia formation, a process with crucial impacts on neuronal development and synapse formation.

## Abstract 2

A novel signaling cascade controlling actin polymerization in response to extracellular signals regulates filopodia formation and likely also neuronal synapse formation.

## Author Summary

Neurons communicate with each other via specialized cell–cell junctions called synapses. The proper formation of synapses (“synaptogenesis”) is crucial to the development of the nervous system, but the molecular pathways that regulate this process are not fully understood. External cues, such as brain-derived neurotrophic factor (BDNF), trigger synaptogenesis by promoting the formation of axonal filopodia, thin extensions projecting outward from a growing axon. Filopodia are formed by elongation of actin filaments, a process that is regulated by a complex set of actin-binding proteins. Here, we reveal a novel molecular circuit underlying BDNF-stimulated filopodia formation through the regulated inhibition of actin-capping factor activity. We show that the actin-capping protein Eps8 down-regulates axonal filopodia formation in neurons in the absence of neurotrophic factors. In contrast, in the presence of BDNF, the kinase MAPK becomes activated and phosphorylates Eps8, leading to inhibition of its actin-capping function and stimulation of filopodia formation. Our study, therefore, identifies actin-capping factor inhibition as a critical step in axonal filopodia formation and likely in new synapse formation.

## Introduction

Deciphering the molecular mechanisms by which neurite extension and synaptogenesis occur during brain development is critical to understand the ontogenesis of the nervous system. In addition, the knowledge of the mechanisms through which initial synaptic contacts are established and modified during brain development may also shed light on synaptic remodeling and plasticity occurring in the adult brain. In the last years, evidence has accumulated indicating that filopodia, which are highly motile, narrow, cylindrical extensions emerging from both axons and dendrites, play important roles at initial stages of synaptogenesis [1],[2]. Furthermore, the growth of new filopodia leading to new synaptic contacts has been suggested as a possible mechanism underlying long-term potentiation [1],[3],[4]. Although axonal filopodia have been investigated less systematically, it is now well established that filopodia extending at the tips of axonal growth cones mediate neurite navigation and axonal path finding [5], whereas filopodia emerging from the shaft of distal axonal branches play a pivotal role in synapse formation [6]. Axonal filopodia differ from both the growth cone and dendritic filopodia since, in addition to bundles of filamentous actin, they also contain clusters of synaptic vesicles that undergo exo-endocytotic recycling [7]–[9]. These packages of vesicles move bidirectionally along the axonal shaft and the filopodia axis, and rapidly coalesce at the site of contact with the appropriate target cell to generate mature presynaptic sites [6],[8]. Thus, filopodia emerging from axons are destined to differentiate into future presynaptic sites.

The core structural and dynamic components of filopodia are actin filaments, whose dynamic formation and topological organization are controlled by ensembles of actin-binding proteins. These proteins play different functional roles in regulating actin dynamics, including binding and/or sequestering of actin monomers, nucleation of actin filaments, capping or anticapping of barbed ends, and severing, bundling, and anchoring of F-actin (filamentous actin) [10]–[12]. In order to elongate, filopodia must be protected from capping and becoming cross-linked into bundles [13]. Formins, which promote the linear elongation of actin filaments in a processive fashion, can also effectively compete with cappers [14]–[16] and have therefore emerged as key players in the generation of filopodia [13]. Alternatively, actin-binding proteins, such as Ena/VASP family members, which bind barbed ends, and bundle and elongate actin filaments [16]–[22], have also been implicated in the formation and elongation of filopodia along neurite shaft and growth cone [17],[20],[23],[24]. Remarkably, mice lacking all three Ena/VASP paralogs (ENA, MENA, and VASP) have defective neuritogenesis in the cortex, leading to a block of cortical axon-fiber tract formation. This defect was shown to arise from a failure of cortical neurons to form filopodia [23]. The neuritogenesis defect observed in Ena/MENA/VASP-deficient cortical neurons could be rescued through intrinsic factors, such as mDia2 or Myosin X, or extrinsic factors, such as laminin, that induce filopodia formation [23]. Instead, no evidence has been reported on the possible role of actin-capping proteins in controlling the formation of axonal filopodia in neuronal cells.

Eps8 is the prototype of a family of proteins involved in the transduction of signal from Ras to Rac, leading to actin remodeling [25]. Eps8 directly controls actin dynamics and the architecture of actin structures by capping barbed ends and cross-linking actin filaments, respectively [26],[27]. The barbed-end capping activity of Eps8, which resides in its conserved C-terminal effector domain, is tightly down-regulated within the context of the holoprotein. Binding of Eps8 to one of its interactors, Abi1, relieves this inhibition [28]. Conversely, Eps8 must associate with IRSp53 (insulin receptor tyrosine kinases substrate of 53 kDa, also known as BAIAP2 for binding partner of the brain-specific angiogenesis inhibitor 1) [29]–[31] to efficiently cross-link actin filaments [27]. These multiple actin regulatory roles of Eps8 in vitro are reflected by the observation that in vivo Eps8 is required for optimal actin-based motility, intestinal morphogenesis, and filopodia-like extension [26]–[28]. The role of Eps8 in neuronal cells is, however, still elusive.

Here, we show that Eps8 is enriched in the growth cone and filopodia of developing hippocampal neurons, where it down-regulates axonal filopodia formation through its barbed-end capping activity. We also show that in vivo, the actin-capping activity of the Eps8/Abi1 complex is modulated by MAPK-mediated phosphorylation of the protein at the residues ser624 and thr628, which occurs in response to external cues, such as BDNF, thereby explaining the induction of axonal filopodia by BDNF treatment.

## Results

### Eps8 Controls the Formation of Axonal Filopodia via Its Actin-Capping Activity

Eps8Ls family proteins are widely and concomitantly expressed in most tissues in developing and adult mice [25]. The brain, in which only the expression of Eps8 and Eps8L2, albeit at lower levels, was detected, represents a notable exception [32]. This prompted a more detailed analysis of the functional role of this protein in neuronal cells.

**Figure 1:**
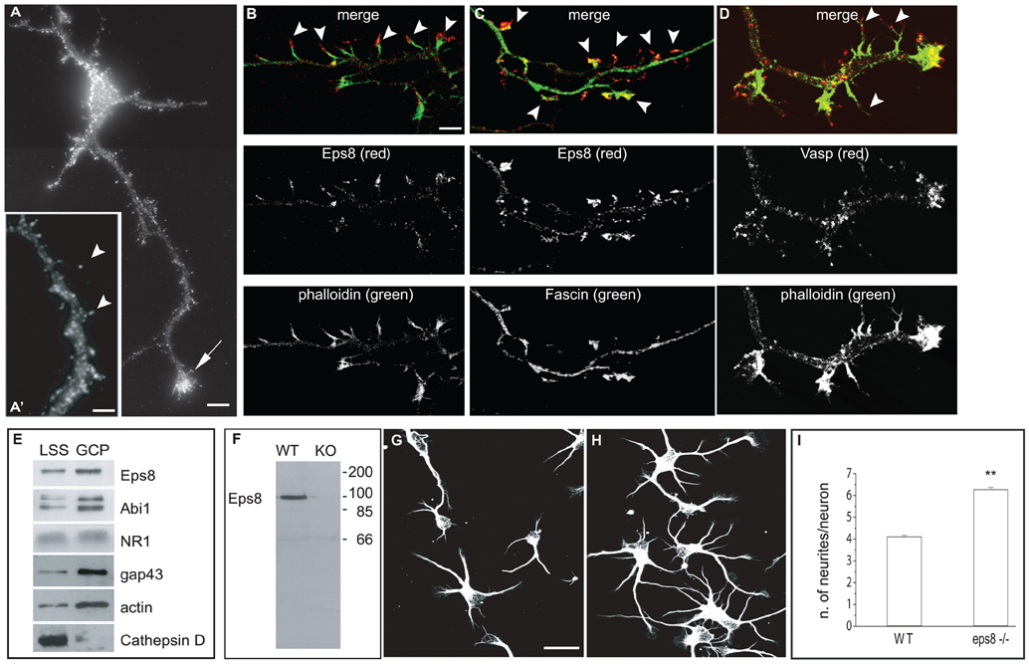
Eps8 is localized to sites of active actin polymerization in hippocampal neurons, and its removal increases neurite number. (A–D) Eps8 localization in hippocampal neurons. Three days in vitro (3DIV) cultured hippocampal neurons were stained with anti-Eps8 antibody alone (A and A′) or in combination with either FITC-conjugated phalloidin to label F-actin (B), or anti-fascin (C) as indicated. The arrow in (A) indicates the axonal growth cone. In the inset (A′), the arrowheads indicate axonal protrusions (arrowheads). The arrowheads indicate Eps8- and actin- (B), or Eps8- and fascin-rich (C) filopodia protruding from the axon. (D) Actin rich axonal protrusions are also immunopositive for VASP. (E) Eps8, its binding partner Abi1, and the growth cone markers GAP43 and actin, are enriched in preparations of growth cone particles (GCPs) relative to low-speed supernatant brain homogenates (LSS). GCP preparations, described in Materials and Methods, were immunoblotted with the indicated antibodies. The immunoblot is a representative example of five independent experiments. (F) Lysates from wild-type (WT) and *eps8* *^−^* ^*/−*^ (KO) hippocampi were immunoblotted with anti-Eps8 antibody. Molecular weight markers are indicated. (G and H) Cultured hippocampal neurons (1DIV) prepared from WT (G) or *eps8* null (H) mice were fixed and stained with anti–beta-tubulin antibody. (I) Quantification of the number of neurites/neuron. Hippocampal neurons were prepared as described in (G and H). The difference between the numbers of neurites of *eps8* *^−^* ^*/−*^ WT neurons is statistically significant (Mann-Whitney rank sum test, *p*≤0.001). Data are expressed as mean±standard deviation (SD); *n* = 897 examined WT neurons, and 760 examined *eps8* KO neurons. Bars indicate 5 µm for (A, B, C, and D); 3 µm for a′; and 10 µm for (G and H). Double asterisks (**) in (F and G) indicate *p*<0.001.

**Figure 2:**
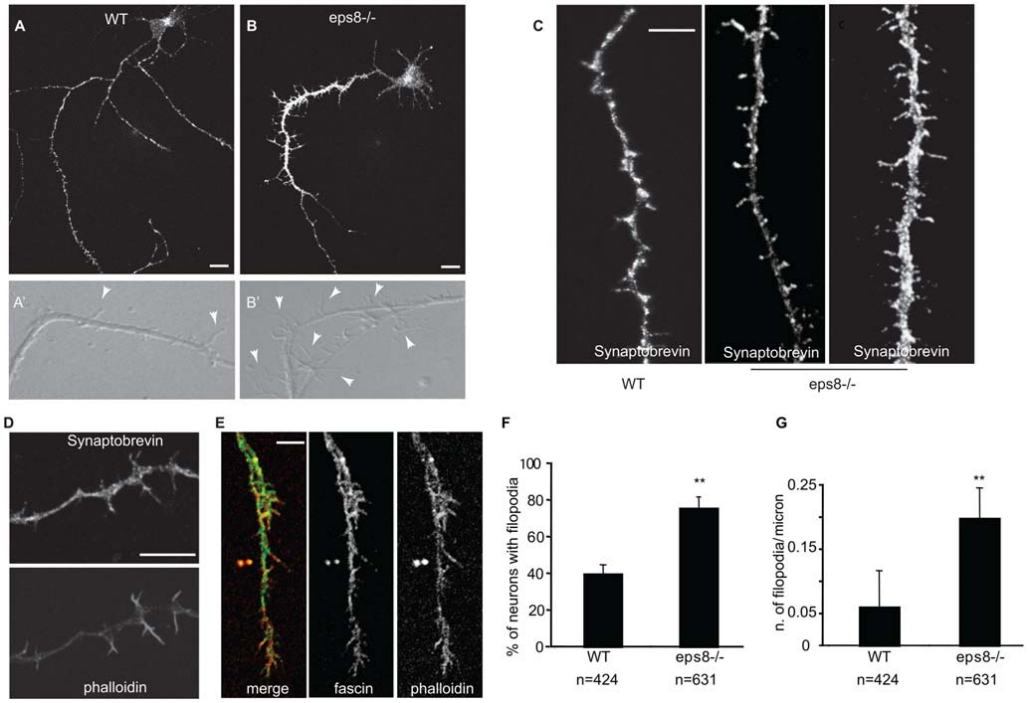
Genetic removal of *eps8* causes increased number of axonal filopodia in hippocampal neurons. (A and B) Cultured hippocampal neurons (3DIV) from WT and *eps8* *^−^* ^*/−*^ mice were fixed and stained with synaptobrevin/VAMP2 antibody. The selective sorting of synaptobrevin/VAMP2 immunoreactivity to a single process reveals that only one neurite is the putative axon. Bar indicates 10 µm. (A′ and B′) Still images of phase-contrast videos of the axonal shaft of cultured hippocampal neurons (3DIV) from WT and *eps8* *^−^* ^*/−*^ mice (see Video S1). Arrowheads highlight axonal filopodia. (C) Representative examples of axonal shafts of WT and *eps8* *^−^* ^*/−*^ neurons, labeled with an antibody against synaptobrevin/VAMP2. Bar indicates 10 µm. (D and E) Filopodia protruding from axons were costained with anti-synaptobrevin/VAMP2 antibody and phalloidin (D) to detect synaptic vesicle and filamentous actin, respectively, or with anti-fascin and phalloidin (E), as indicated. Bar indicates 7 µm. (F and G) Neurons from *eps8* *^−^* ^*/−*^ mice display a significantly higher density of axonal filopodia. (F) Quantification of the percentage of neurons with filopodia in WT and *eps8* *^−^* ^*/−*^ neurons. Neurons with more than 0.04 filopodia/µm were considered as filopodia-bearing neurons (Student *t*-test, *p*≤0.001). (G) Quantification of the density of axonal filopodia in WT and *eps8* null hippocampal neurons (Mann-Whitney rank sum test, *p*≤0.001). *n* = 424 examined WT neurons and 621 examined *eps8* KO neurons. Data are expressed as the mean±SD.

In 3–4-d-old primary hippocampal cultures, we found Eps8 expressed in neuronal cell body and neurites, and prominently enriched in the axonal growth cone (Figure 1A, arrow). In both rat (Figure 1A–1C) and mouse (unpublished data) hippocampal neurons, Eps8 was also detected along finger-like protrusions emerging from the axonal shaft. These processes measured 3.27 µm±0.07 standard error (SE) (in line with [33]), were highly dynamic (Video S1), contained actin filaments (Figure 1B and 1D, see also [7],[9]) and the actin-binding proteins fascin (Figure 1C) and VASP (Figure 1D), and were characterized by the presence of synaptobrevin/VAMP2-positive vesicle clusters (Figure 2C and 2D, see also [8],[9]), thus they are bona fide axonal filopodia.

Immunoblotting of subcellular fractions of fetal rat cortices further indicated that Eps8 and its binding partner Abi1 were concentrated in growth cone particles (GCPs) (Figure 1E), a preparation that allows the investigation, using a biochemical approach, of the expression and interaction of growth cone and axonal proteins during neuronal development [34],[35]. GCPs are enriched in pinched-off, resealed, metabolically active growth cones [34],[35], as witnessed by the presence of the growth cone–specific marker GAP43 and of actin [36],[37]. Conversely, the NMDA receptor subunit NR1, which is uniformly distributed in the neuronal plasma membrane at this developmental stage [38], is not enriched in the GCPs, whereas the proteolytic enzyme cathepsin D, as expected, is excluded from this fraction, consistent with its prevalent localization in the neuronal soma (Figure 1E).

To investigate the functional role of Eps8 in neuronal cells, we established primary hippocampal cultures from *eps8* WT and deficient mice, and analyzed their morphology (Figure 1F–1I). Strikingly, when compared to age-matched WT, *eps8* ***^−^*** ^*/****−***^ neurons displayed a significantly higher number of processes emerging directly from the cell body (primary processes) (Figure 1G–1I). Closer inspection of these cultures revealed that *eps8* ***^−^*** ^*/****−***^ neurons properly formed one single axon, positive for synaptobrevin/VAMP2, which is sorted to the axon at early stages of neuronal development (Figure 2A and 2B). Conversely, a significantly higher number of axonal filopodia was evident in *eps8* null when compared to control WT neurons (Figure 2A, 2A′, 2B, 2B′, and 2C, and Video S1). These protrusions contained synaptic vesicle clusters (Figure 2C and 2D), were enriched in F-actin (Figure 2D and 2E), were immunoreactive for fascin (Figure 2E), and were significantly reduced by functional interference with VASP using a dominant-negative approach [17] (Figure S1). Both the percentage of neurons displaying filopodia emerging from the axons and the density of axonal filopodia were increased by either genetic (Figure 2F and 2G) or RNA interference (RNAi)-mediated ablation of Eps8 (Figure S2A–S1E). On the other hand, no effect on filopodia length was observed (unpublished data). Finally, dendritic filopodia also were augmented in *eps8* ***^−^*** ^*/****−***^ neurons at early stages of dendrite formation (7 d in vitro [7DIV]) (Figures S2F and S1G). Thus, Eps8 is critical for the proper extension of neuronal filopodia, and its removal in neurons increases the formation of axonal filopodia.

**Figure 3:**
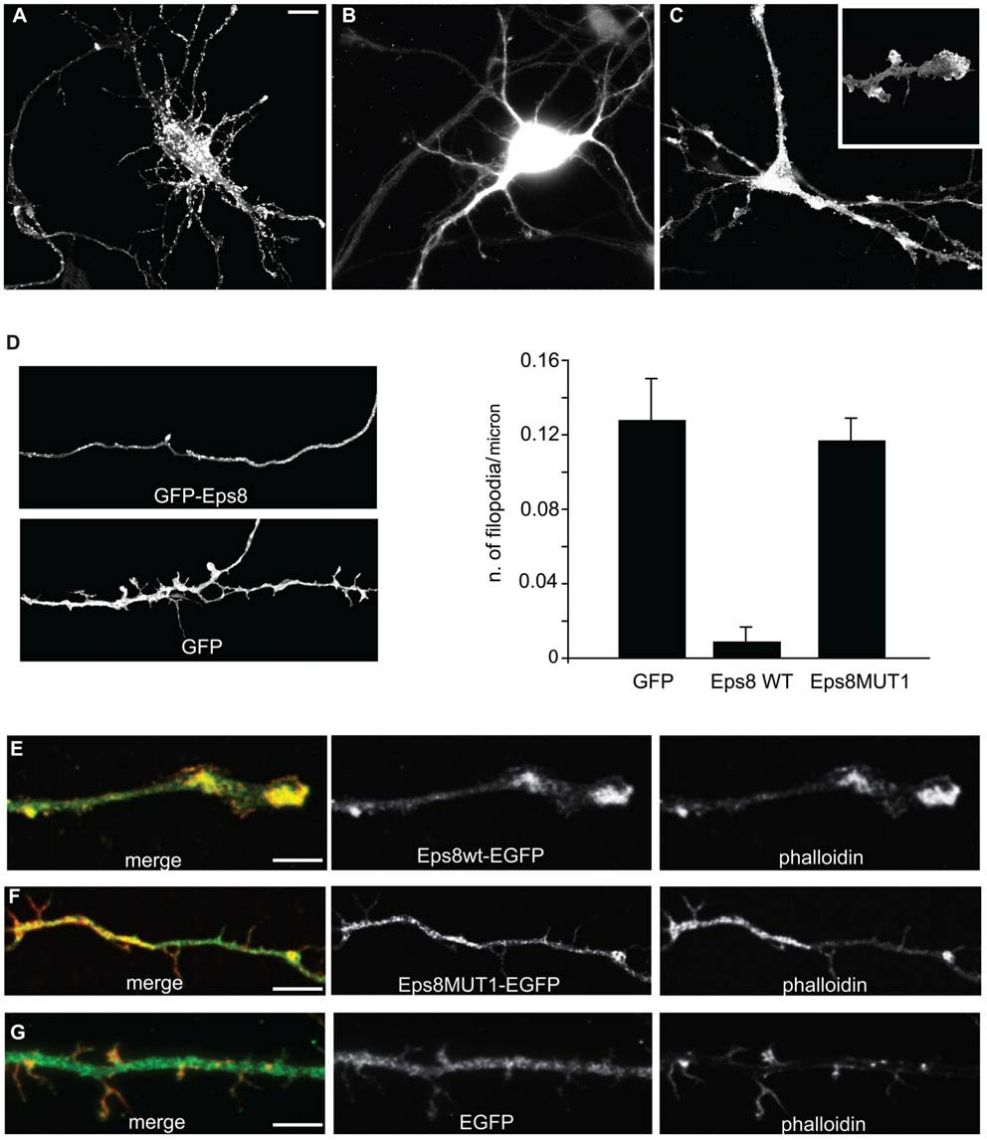
The regulation of filopodia density depends on Eps8 actin barbed-end capping activity. (A–D) Primary *eps8* *^−^* ^*/−*^ hippocampal neurons (3DIV) were infected with either WT Eps8-EGFP (A and C) or control EGFP (B) lentiviral vectors, then fixed and processed for epifluorescence. Neurons expressing Eps8 ([D] left top panel), but not those expressing EGFP ([D] left bottom panel), show a reduction in the density of axonal filopodia ([D] right panel; number of examined neurons: 120 Eps8wt-EGFP, 55 EGFP; Kruskal-Wallis one-way analysis of variance, *p*<0.001) Data are expressed as the mean±SEM. Of note, occasionally high levels of ectopically expressed Eps8 induced the presence of flattened structures, sometimes with club-like endings, ([C] and inset in [E]). (E–G) Primary *eps8* *^−^* ^*/−*^ hippocampal neurons (3DIV) infected with either Eps8wt-EGFP (E) or Eps8MUT1-EGFP (number of examined neurons: 31 [F]), an Eps8 mutant devoid of actin-capping activity (Figure S3), or control EGFP (G) lentiviral vectors were fixed and processed for epifluorescence or stained with Texas Red phalloidin, as indicated. Merged images are also shown. Eps8MUT1 neurons do not display flattened membrane protrusions and are morphologically undistinguishable from EGFP-infected neurons (see also quantitation [D], right panel). Bar indicates 10 µm for (A, B, and C); 3 µm for the inset in (C), 5 µm for (D), and 8 µm for (E and G).

Next, we investigated whether the increase of axonal filopodia in *eps8* null neurons depends on the protein actin barbed-end capping or cross-linking activity residing in its conserved carboxy-terminal effector domain [27],[28]. To this aim, we restored Eps8 expression in *eps8* ***^−^*** ^*/****−***^ cultures with a lentiviral vector encoding either a GFP-tagged Eps8 WT or an Eps8 mutant, Eps8MUT1, specifically devoid of actin capping (Figure S3A), but retaining actin-binding and -bundling activity (Figure S3B and S3C). The infection of *eps8* null neurons with the GFP-tagged Eps8 lentiviral vector led to a significant reduction in the number of axonal filopodia to WT levels (Figure 3A–3D). Thus, the filopodia phenotype is specifically caused by the lack or reduction of Eps8.

Conversely, Eps8MUT1-infected neurons were morphologically indistinguishable from GFP-infected cells (compare Figure 3F with 3G, see quantitation in Figure 3D) and retained an increased number of axonal filopodia. It is of note that occasionally, and especially at high levels of ectopic expression of WT Eps8, we observed thick, sometimes club-like actin-based protrusions along both axon and dendrites (Figure 3C and insert, Figure 3E), never detectable in GFP-infected neurons, which showed a morphological appearance comparable to *eps8* ***^−^*** ^*/****−***^ neurons (Figure 3B, 3D, and 3G). Collectively, these results support a critical role of the barbed-end capping activity of Eps8 in regulating the balance between different types of actin-based membrane extensions, and more specifically, in controlling the formation of axonal filopodia protrusions.

Eps8, to function as capper, requires the binding of its interactors Abi1 or Abi2 [28]. Therefore, we analyzed the ability of these latter proteins to form a complex with Eps8 in brain lysates. Both Abi1 and the brain-specific Abi2 coimmunoprecipitated with Eps8 (Figure S4A), indicating the existence of active capping complexes and supporting the importance of the capping activity in the nervous system. Conversely, in epithelioid HeLa cells, no evidence of a complex between Abi1 or Abi2 and Eps8 could be found (Figure S4A), likely due to the fact that either Abi2 is expressed at lower levels than in brain (A, right panel) and/or these proteins are sequestered by interaction with other partners. These latter findings suggest that the actin-capping activity of Eps8 is critical for filopodia in neurons, but not in HeLa cells, as previously shown [27].

Eps8 can form a complex with Abi1, Sos-1, and PI3K [39] able to activate Rac, which, in turn, is involved in the formation of neurites and filopodia-like processes in neurons [40] and is activated by BDNF [41]. Thus, we examined the possibility that the increase in axonal filopodia observed after genetic removal of Eps8 could be due to reduced levels of Rac GTP. To this end, we performed two sets of experiments. In the first approach, we directly tested whether Eps8 removal impaired Rac activation. Measurement of RacGTP levels by affinity-based CRIB assays revealed that equal levels of RacGTP were present in lysates of brain cortex and hippocampus derived from WT and *eps8* null mice (Figure S4B), indicating that in brain, Eps8 is not required for Rac activation. As additional proof, the impairment of Rac activation, which might be predicted to occur as a consequence of Eps8 removal [42], was mimicked by exposing hippocampal neurons to the specific Rac1 inhibitor, NSC23766 [43]. We demonstrated the efficacy of this compound by showing that NSC23766 administration (1) prevented PDGF-induced actin ruffles in NIH3T3 cells (Figure S4C), a well-known process involving Rac activation [44], and (2) decreased RacGTP levels of 3DIV hippocampal cultures (Figure S4D). Notably, in hippocampal neurons, a reduction rather than an increase in the number of filopodia occurred upon Rac inhibition (Figure S4E), as previously shown [45]. Thus, the pathways leading to Rac activation are not the underlying molecular cause of the enhancement of axonal filopodia extension observed in *eps8* null hippocampal neurons [45].

### BDNF Induces Filopodia Formation via MAPK Activation in Wild-Type, but Not *eps8* ***^−^*** ^*/****−***^ Neurons

**Figure 4:**
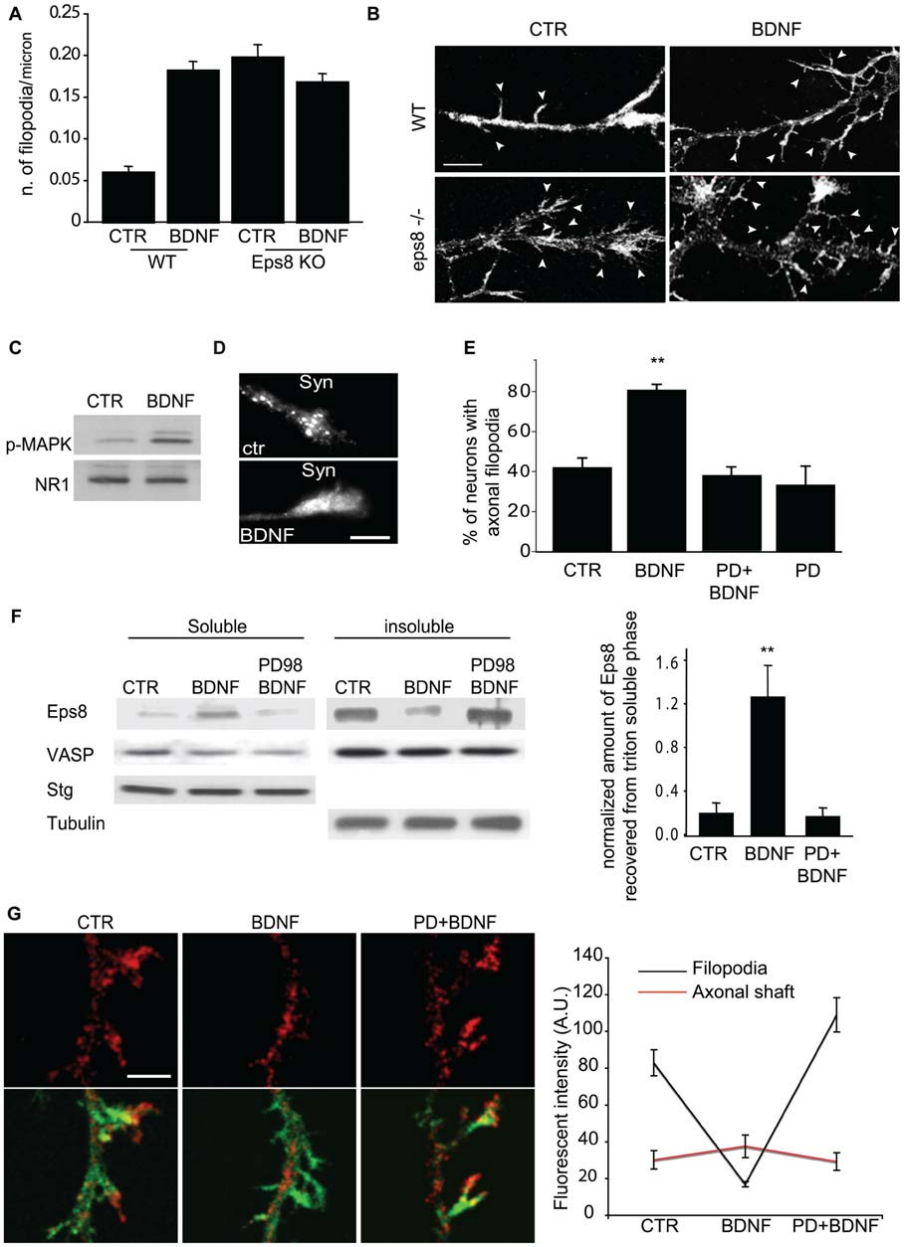
BDNF induces filopodia and regulates Eps8 intracellular localization through MAPK activation. (A and B) BDNF fails to increase the number of filopodia in *eps8* *^−^* ^*/−*^ neurons. Wild-type (WT) and *eps8* *^−^* ^*/−*^ (KO) hippocampal neurons (3DIV) treated with BDNF (100 ng/ml for 20 min) or mock-treated (CTR), were fixed and stained to detect F-actin (B). Arrowheads highlight axonal filopodia. The density of filopodia was calculated as in Figure 2F. Data are expressed as the mean±SD. Kruskal-Wallis one-way ANOVA on ranks, Dunn's methods for comparison among groups: there is a significant difference between “WT Ctr” and “WT BDNF” (*p*<0.05), whereas there is no difference among “WT BDNF”, “eps8 KO Ctr,” and “eps8 KO BDNF” (*p*>0.05). (B) Example of axonal filopodia induced by BDNF in WT and *eps8* *^−^* ^*/−*^ hippocampal neurons fixed and stained as described above. Bar indicates 5 µm. (C) Lysates of GCPs treated with BDNF (100 ng/ml for 20 min) were immunoblotted with the indicated antibodies to detect phosphorylated MAPK and NR1 subunit of NMDA receptor, used as loading control. (D) Hippocampal neurons treated with BDNF (100 ng/ml for 20 min) (BDNF) or mock-treated (CTR) were stained with anti-Synapsin I antibody (Syn). Bar indicates 2 µm. (E) Hippocampal neurons were incubated with PD98059 or vehicle as control, and treated with BDNF (100 ng/ml for 20 min) (BDNF) or mock-treated (CTR). Cells were fixed and processed as above. Neurons with axonal filopodia (neurons with more than 0.04 filopodia/µm were considered as positive) were quantified. BDNF treatment almost doubles the percentage of neurons with filopodia, whereas pretreatment with PD98059 prevents this effect (one-way ANOVA, post hoc Tukey test, double asterisks [**] indicate *p*≤0.001). Data are expressed as the mean±SD. (F) GCPs treated (BDNF) or not (CTR) with BDNF, prior or not PD98059 exposure, were fractionated using 1% Triton X100. Triton X100–soluble (sol) and -insoluble (ins) fractions were immunoblotted with the indicated antibodies. Synaptotagmin I (Stg) and tubulin were used as controls. Right graph: quantification of the Eps8 distribution to Triton X100–soluble fraction was performed by densitometry and normalized using synaptotagmin I (one-way ANOVA, post hoc Tukey test, *p*≤0.001). (G) Left: vehicle or PD98059-incubated hippocampal neurons (3DIV) were mock-treated or treated for 20 min with 100 ng/ml BDNF, as indicated. Cells were fixed and stained with anti-Eps8 antibody (red) and FITC-phalloidin to detect F-actin (green). Merged images are shown. Note that Eps8 immunoreactivity is virtually lost from filopodia upon BDNF treatment and that PD98059 prevents this relocalization. Right graph: quantitation of Eps8 fluorescence intensity measured in filopodia and axonal shafts under the different experimental conditions. Error bars represent SEM. Quantitation has been carried out by ImageJ software on 30 images from three independent experiments, filopodia are identified by labeling with phalloidin (one-way ANOVA, *p*<0.001 in filopodia and *p*>0.05 in axonal shaft). Bar indicates 5 µm. Note that Eps8 immunoreactivity is significantly reduced from filopodia upon BDNF treatment, and restored by PD98059.

Having established a critical role for Eps8 capping activity in down-regulating the formation of axonal filopodia, we next investigated how Eps8 biological functions might be regulated in vivo. Eps8 was originally isolated as a substrate of receptor tyrosine kinases activated in response to growth factors [46]. Therefore, we focused on the neurotrophic factor BDNF, which critically controls growth and differentiation processes in the brain during development, through the activation of Trk tyrosine kinase receptors. Consistent with this, and as described previously [47], treatment of hippocampal neurons with BDNF induced the formation of axonal actin- and fascin-rich filopodia, mimicking genetic and RNAi-mediated *eps8* removal (Figure 4A and 4B). Remarkably, however, the BDNF-induced increase in filopodia density was not detected in *eps8* ***^−^*** ^*/****−***^ cultures (Figure 4A and 4B). Even if we cannot exclude the possibility that BDNF has no effect on filopodia in *eps8* ***^−^*** ^*/****−***^ neurons simply because the density of filopodia is already maximal, this observation points to a role of Eps8 in BDNF-mediated filopodia formation. BDNF induced, as expected, MAPK activation in the GCPs (Figure 4C), and MAPK-dependent phosphorylation of the synaptic vesicle protein synapsin I [37], leading to its dispersion in the distal axonal shaft (Figure 4D) [37],[48]. More importantly, the formation of filopodia induced by the neurotrophin was inhibited by pretreatment of the cultures with the specific MAPK inhibitor, PD98059 (Figure 4E), indicating that MAPK activation is also critical in this pathway.

### BDNF Controls the Subcellular Distribution of Eps8 via MAPK Phosphorylation

One mechanism to control actin dynamics in response to extracellular cues is to ensure the correct, spatially restricted localization of critical actin-binding proteins, such as Eps8 and its interactors. We thus examined the distribution of Eps8 to the actin-rich, Triton X100–insoluble fraction of GCPs in response to BDNF. Treatment with BDNF increased the amounts of Eps8 (Figure 4F) and, slightly, of its activator Abi1 (unpublished data), recovered in the Triton X100–soluble fraction, causing a reciprocal decrease in the Triton X100–insoluble fraction (Figure 4F). Conversely, VASP was mainly found in the Triton-insoluble fraction, and its distribution was unaffected by BDNF stimulation (Figure 4F). Finally, Eps8 redistribution to the Triton X100–soluble fraction correlated with BDNF-induced cellular relocalization of Eps8, which disappeared from filopodia, persisting instead along the axonal shaft, upon BDNF stimulation (Figure 4G and quantification). Notably, both Eps8 redistribution to the Triton-X100-soluble fraction and cellular relocalization by BDNF were prevented by preincubation with the MAPK inhibitor, PD98059 (Figure 4F and 4G). Thus, collectively, these results indicate that BDNF controls, in a MAPK-dependent manner, the dynamic cellular redistribution of Eps8 in vivo.

**Figure 5:**
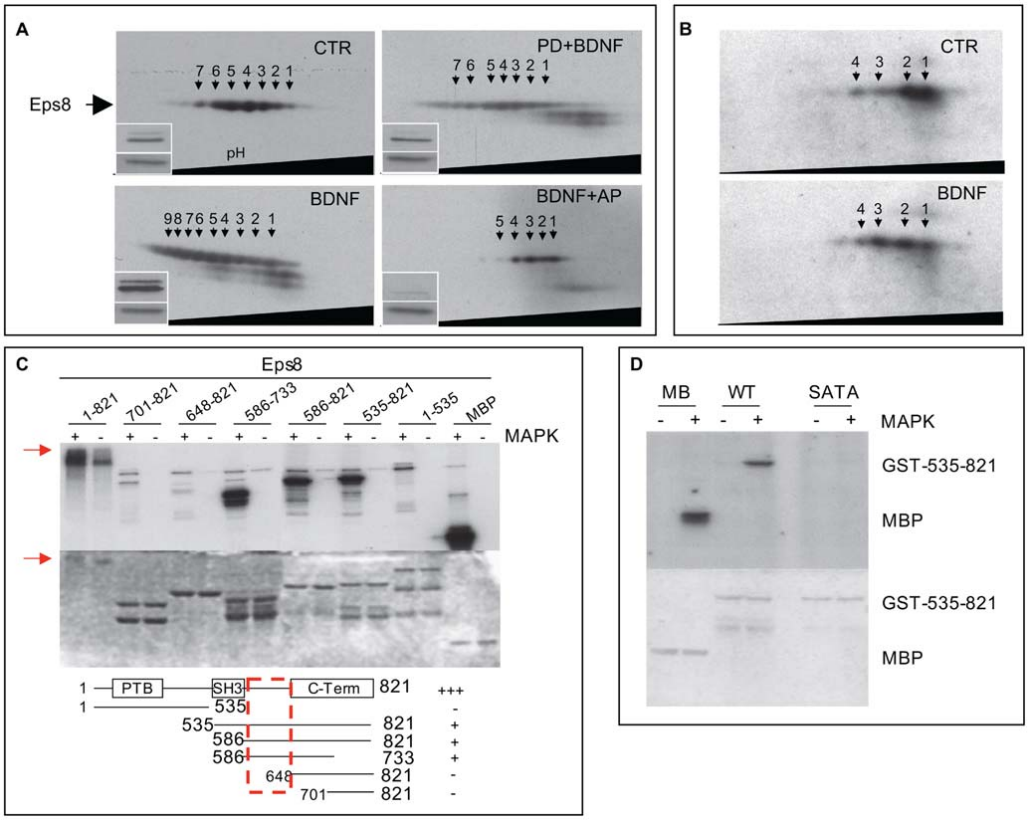
Eps8 is phosphorylated by MAPK. (A) 2-D immunoblot analysis of Eps8 from synaptosomes mock-treated (CTR) or treated with BDNF (BDNF) or pretreated with PD98059 before BDNF stimulation (PD+BDNF), or treated with BDNF followed by incubation with alkaline phosphatase (BDNF+AP). Note the appearance, upon BDNF application, of additional and more intense Eps8 immunopositive spots with more acidic isoelectric points (spots: 6–9). These spots disappear by preincubating synaptosomes with alkaline phosphatase. Insets show the analysis of synaptosomes treated as above, resolved by monodimensional SDS-PAGE and immunoblotted with anti–phospho-MAPK (upper band) or anti-ribophorin (lower band), the latter used as loading control. (B) 2-D immunoblot analysis of Eps8 in GCPs in the absence (CTR) or the presence of BDNF. (C) Recombinant purified His-Eps8 (1–821) or recombinant GST-fused Eps8 fragments (5 µg) were incubated with 10 µCi [γ-32P]-ATP in the presence (+) or absence (−) of purified MAPK (20 ng) in kinase buffer (see Materials and Methods), resolved by SDS-PAGE, stained with Coomassie Blue (lower panel), and subjected to autoradiography (upper panel). The red arrow indicates the shift of Eps8 due to phosphorylation. (D) Recombinant purified GST-Eps8 535–821 wild-type (WT) or S624A, T628A (SATA) mutant (5 µg) were incubated with 10 µCi [γ-32P]-ATP in the presence (+) or absence (−) of purified MAPK (20 ng) in kinase buffer (MB), resolved by SDS-PAGE, stained with Coomassie Blue (lower panel) and subjected to autoradiography (upper panel). In (C and D), MBP (myelin basic protein) was used as a control substrate.

To investigate the molecular mechanisms through which BDNF and MAPK regulate Eps8, its posttranslational modifications in synaptosomes were analyzed by two-dimensional electrophoresis. Seven Eps8 spots of similar molecular size, but different isoelectric points (IP, Figure 5A, CTR), were detected by immunoblotting with two independently raised antibodies (affinity-purified polyclonal antibody, Figure 5A; monoclonal antibody, unpublished data), suggesting the existence of posttranslational modified forms of Eps8. Competition of Eps8 monoclonal and polyclonal antibodies with the respective immunogenic polypeptide further validated the identity of the immunoreactive spots (unpublished data). More importantly, treatment of synaptosomes with BDNF induced a change in the electrophoretic pattern of Eps8 with the appearance of additional spots of increased acidic IP (spots 6–9 in Figure 5A, BDNF), which were significantly diminished by pretreatment with MAPK inhibitor PD98059 prior to BDNF stimulation (Figure 5A, PD+BDNF). Additionally, the five most acidic spots of Eps8 could no longer be detected after dephosphorylation of synaptosomes with alkaline phosphatase (Figure 5A, BDNF+AP). Similar results were obtained in preparations of GCPs (Figure 5B). Of note, Eps8 shows a different electrophoretic pattern in untreated GCPs with respect to untreated synaptosomes (Figure 5B, “CTR,” and 5A, “CTR”), suggesting that the protein is subjected to different posttranslational modifications during brain development. Remarkably, however, and at variance with stimulation with other RTK [46], we could detect no Eps8 tyrosine phosphorylation after BDNF treatment of synaptosomes (Figure S4F). Thus, collectively, the above results support the notion that MAPK or a MAPK-dependent distal kinase is responsible for Eps8 phosphorylation in response to BDNF. To directly assess this possibility, we carried out an in vitro phosphorylation assay. Purified Eps8 became phosphorylated by recombinant purified MAPK to a similar extent as a bona fide MAPK kinase substrate (myelin basic protein [MBP]) (Figure 5C, first two lanes, and Figure S5A), thus indicating that Eps8 is a direct substrate of activated MAPK.

### Phosphorylation of S624 and T628 Controls the Barbed-End Capping Activity of the Eps8:Abi1 Complex and Eps8 Subcellular Relocalization in Response to BDNF

To get insights into the residues specifically phosphorylated by MAPK, a structure–function analysis was carried out using different fragments of Eps8. Eps8 (586–733) is the minimal fragment that is phosphorylated (Figure 5C). However, the lack of phosphorylation of Eps8 (648–821) restricted further the phosphoresidue-containing region of Eps8 to amino acids 586–648. This region contains two putative consensus sites (S624 and T628) for MAPK (Prosite [49], Scansite [50]). Simultaneous mutation of these sites to A (GST-535-821-SATA), either in the context of the C-terminal 535–821 fragment of Eps8 (Figure 5D) or in the full-length protein (Figure S5A), abrogated MAPK-mediated phosphorylation, unequivocally identifying the Eps8 residues targeted by MAPK.

**Figure 6:**
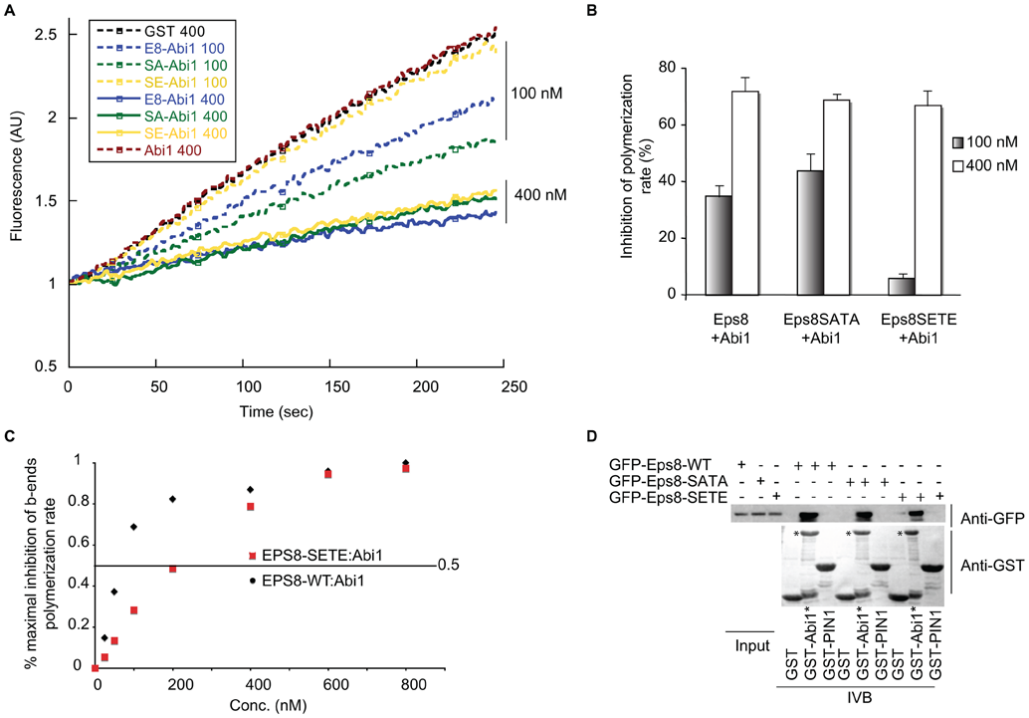
The phosphomimetic Eps8 (Eps8-SETE) mutant has a 10-fold reduced barbed-end capping activity with respect to Eps8 WT or phosphoimpaired Eps8 (Eps8-SATA) mutant when in complex with Abi1. (A) The indicated, increasing concentrations of equimolar amounts of purified His–Eps8 WT, SATA, and SETE (Eps8, SATA, and SETE) and GST-Abi1 were incubated with 2.5 µM G-actin (10% pyrenyl-labeled) in polymerization buffer. Actin polymerization was initiated by spectrin–actin seeds as described in Materials and Methods. GST was used as a control. (B) The relative inhibition of barbed-end polymerization rate at the indicated concentrations of the Eps8-WT:Abi1, Eps8-SATA:Abi1, and Eps8-SETE:Abi1 complexes is shown. Under suboptimal, nonsaturating conditions (100 nM concentrations), the Eps8-SETE:Abi1 complex is less effective in inhibiting barbed-end growth than Eps8 WT or Eps8-SATA:Abi1 complexes. Three independent experiments were performed. Error bars indicate SEM. (C) The relative inhibition of barbed-end polymerization at the various, indicated concentrations of the WT Eps8:Abi1 or Eps8-SETE:Abi1 complexes. The concentrations of Eps8-WT:Abi1 and Eps8-SETE:Abi1 at which half-maximal inhibition (IC_50_) was observed were 60 and 200 nM, respectively, which correspond to a concentration of the respective complexes of: Eps8(WT):Abi1 = 0.15 nM; Eps8-SETE:Abi1 = 1.5 nM (based on calculated thermodynamic constant of association between Eps8 and Abi1 (*K*_d_ = 35 µM [72]). The data shown are representative of four independent experiments with similar results. (D) Eps8 phosphomutants bind Abi1 similar to Eps8 WT. Lysates of cells expressing GFP-Eps8 WT or SATA or SETE (indicated at the top) were incubated with immobilized GST, or GST-PIN1, used as negative control, or GST-Abi1 (asterisk [*] in the figure). The lower bands on the GST-Abi1 lanes are likely premature termination or degradative products of the Abi1 moiety of the fusion protein. Lysates and bound proteins were immunoblotted with the indicated antibodies (on the right). IVB, in vitro bound.

To demonstrate the biochemical and physiological relevance of these phosphosites, we generated phosphoimpaired and phosphomimetic mutants of full-length Eps8 by substituting S624 and T628 with either A (Eps8-SATA) or E (Eps8-SETE), respectively. We initially tested the effects of these mutations using in vitro assays of actin polymerization either in the context of the isolated C-terminal, constitutive active Eps8 (535–821) fragment, or with full-length Eps8 protein, whose activity is unmasked when in complex with Abi1 [28]. We detected no difference in the affinities for barbed ends when WT and Eps8 (535–821) mutants were compared, suggesting that these phosphoresidues do not interfere with the direct binding of the isolated Eps8 capping domain to actin barbed ends (Figure S5B and S5C). Of note, phosphorylation of S624 and T628 did not affect the actin filaments side-binding capacity of Eps8 (Figure S5D and S5E). Thus, when Eps8 C-terminal fragments are folded into a presumably “open” conformation that allows its association to actin barbed ends and filaments, we could evidence no detectable effects of its phosphorylation. Conversely, the affinity of the Eps8-SETE:Abi1 complex for actin filament barbed ends was about 10 times lower that of the Eps8-WT:Abi1 or EPS8-SATA:Abi1 complexes (Figure 6A–6C). Importantly, Eps8-SATA and -SETE bound Abi1 with similar affinities as Eps8WT (Figure 6D). Thus, phosphorylation of Eps8 significantly impaired the actin-capping activity of Eps8:Abi1 complex, most likely by facilitating Abi1's ability to promote the structural changes required to activate Eps8.

**Figure 7:**
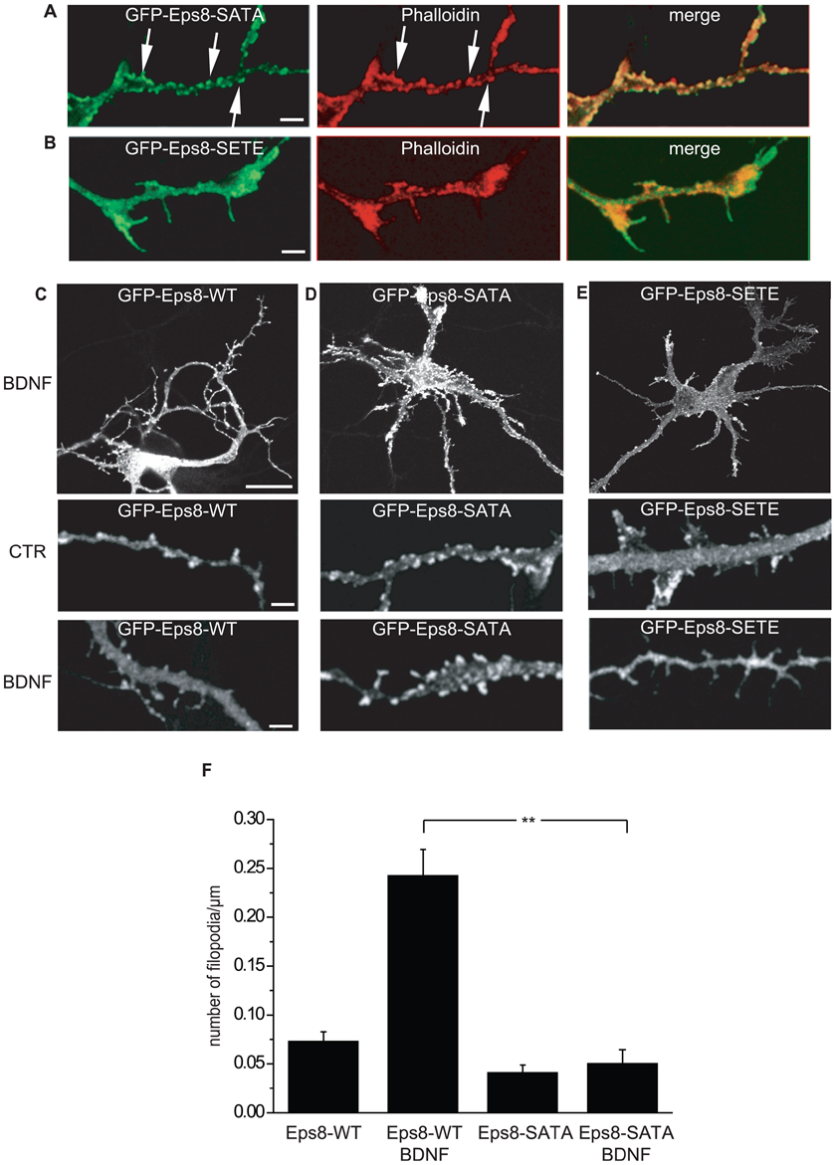
Eps8-SATA is preferentially localized along actin-rich structures and inhibits BDNF-induced filopodia. (A and B) Hippocampal neurons (3DIV) were transfected by calcium phosphate with GFP-Eps8-SATA (A), GFP-Eps8-SETE (B) and examined 20 h after transfection. Cells were fixed and stained with TRITC phalloidin (red) and processed for epifluorescence to detect F-actin and Eps8 (green), respectively. Merged images are shown. Bar indicates 5 µm. (C–E) BDNF induces dispersion of WT Eps8, but not of Eps8-SATA or Eps8-SETE. Hippocampal neurons (3DIV) transfected with GFP-Eps8-WT (C), GFP-Eps8-SATA (D), or GFP-Eps8-SETE (E) were examined 20 h after transfection, upon stimulation with 100 ng/ml BDNF (BDNF) or mock-treatment (CTR). Cells were fixed and processed for epifluorescence as described above. Bar indicates 10 µm in the upper panels and 5 µm in the magnified axons shown in the lower panels. (F) Quantification of the density of filopodia in cultured hippocampal neurons (3DIV) transfected with GFP-Eps8-WT (*n* = 26) or GFP-Eps8-SATA (*n* = 34) and analyzed 20 h after transfection, either untreated or upon 20-min exposure to BDNF (100 ng/ml). Data are expressed as the mean±SD (Student *t*-test, double asterisks [**] indicate *p*≤0.001).

Next, we tested whether these posttranslational modifications of Eps8 had any effect on Eps8 distribution, association to actin-based structures, or BDNF-mediated relocalization. Remarkably, we found that in neuronal cells, Eps8-SATA was distributed to and colocalized with discrete actin-rich structures (Figure 7A), whereas Eps8-SETE showed a more diffuse distribution (Figure 7B), in line with the hypothesis that the latter, phosphomimetic construct is constitutively detached from actin filaments. Additionally, and more importantly, WT Eps8 (Figure 7C), but not Eps8-SATA (Figure 7D), was dispersed after BDNF stimulation. This is consistent with the possibility that, being unable to be phosphorylated, Eps8-SATA does not detach from actin even after BDNF treatment. Finally, the expression of Eps8-SATA (Figure 7D and 7G), but not Eps8-SETE (Figure 7E), significantly inhibited the formation of BDNF-induced axonal filopodia (Figure 7G), acting in a dominant fashion. Collectively, these data indicate that phosphorylation of S624, T628 residues controls the barbed-end capping activity of the Eps8:Abi1 complex and Eps8 subcellular localization in response to BDNF. Thus, one mechanism to activate the protrusive activity by BDNF in neurons is to down-modulate the activity of capping proteins, via MAPK phosphorylation, favoring actin filament elongation and axonal filopodia extensions.

## Discussion

In this study, we demonstrated through genetic and cellular biochemical approaches, a crucial role of the actin barbed-end capping activity of Eps8 in down-regulating the formation of VASP-dependent neuronal filopodia. We further show that this activity is controlled by phosphorylation of residues S624 and T628 of Eps8, which occurs upon MAPK activation in response to BDNF, thus unraveling the molecular components of a novel signaling cascade converging on a capping protein and ultimately leading to the proper formation of axonal filopodia.

Filopodia are formed by cross-linked actin filaments, whose initiation is controlled by the balance between capping and anticapping activities. Anticapping activity can be executed either by formins, which promote linear elongation of actin filaments from barbed ends competing with cappers, or by Ena/VASP proteins, albeit through multiple and diverse biochemical mechanisms. Indeed, VASP family proteins may directly [17],[18] or indirectly antagonize capping proteins [22], capture barbed ends [51], and cross-link actin filaments [20],[22]. Formins and Ena/VASP family proteins act independently of each other, as demonstrated by the finding that genetic removal of Ena, MENA, and VASP abrogates filopodia, which, however, can be restored by expression of the formin mDia2 [23]. Thus, there are likely multiple and independent mechanisms that give rise to filopodia. This is reflected by the fact that different models have been proposed to account for the initiation and elongation of filopodia with respect to other protrusions, such as lamellipodia. For instance, the convergent elongation model [52] proposes that the Arp2/3-dependent dendritic nucleation of actin filaments is the starting point of actin filament arrays in both lamellipodia and filopodia. The activity of capping proteins, according to this model, is then crucial to control the subsequent architectural organization of actin in these protrusions. When the capping activity is high, newly nucleated branched filaments become rapidly capped. The balance between de novo branched nucleation and elongation by Arp2/3 and blockade of filament growth by capping proteins, both competing for filament ends, would thus result in the generation of a dendritic actin network at the leading edge of lamellipodia. In this context, it is remarkable that Eps8, when expressed at high levels, causes the formation of flat, actin-rich protrusions along axons, which resemble lamellipodia extensions (Figure 3). Conversely, when capping activity is low, such as after RNAi-mediated interference or genetic knockdown of Eps8 in neurons, filaments nucleated by Arp2/3 [53] can grow longer, uncapped. Factors such as VASP family members or components of the filopodia tip complex may then promote the transient association of actin filaments, which can be further stabilized by other cross-linkers, such as fascin, thus permitting the formation of bundles of sufficient stiffness to overcome buckling and membrane resilience [54]. Of note, filopodia initiation, rather than elongation, is affected by Eps8. A corollary of this model is that Eps8-dependent filopodia should be, at least in part, mediated by VASP. Consistently, we show here that the increased axonal filopodia of *eps8* null hippocampal neurons are significantly reduced upon interference of VASP functions through dominant-negative approaches, suggesting functional competition between the capping activity of Eps8 and the actin-regulatory properties of VASP. This is reminiscent of a situation similar to the one suggested by Mejillano and colleagues in nonneuronal cells, where depletion of the capping protein CP caused loss of lamellipodia and the explosive formation of filopodia, a phenotype which is counteracted by Ena/VASP proteins [21].

An alternative model of filopodia initiation questioned the relevance of Arp2/3-mediated nucleation (reviewed in [13]), claiming instead that linear filament elongation at the leading edge of protrusions is the source of bundles in filopodia. Within this context, formins [16],[55]–[57] or clustered VASP proteins [22] may be the culprits in promoting filopodia initiation. Also in this case, however, filaments must be protected from cappers, which have been shown, in the case of CP, to compete, either directly or indirectly, with VASP as well as with formins for barbed-end binding. Whether Eps8 can compete with clustered VASP or formins for barbed ends remains to be assessed. The fact that filopodia induced by removal of Eps8 are dependent on VASP argues, however, that a balanced activity of these two proteins may be critical for proper axonal filopodia formation.

Notably, Eps8 is a multifunctional protein, whose actin-related activities, capping and bundling, are critically controlled by its binding partners, Abi1 and IRSp53, respectively [27],[28]. Both capping and bundling activities need to be coordinated and regulated for the initiation and maintenance of filopodia. In neurons, in which Eps8 is tightly bound to Abi1 or Abi2, the capping function prevails, exerting a negative modulatory role on filopodia extension. Conversely, in HeLa cells, where the majority of Eps8 is associated with IRSp53 [27], and no complex with Abi1 or Abi2 is detected, the Eps8:IRSp53 complex bundles actin filaments, positively contributing to the formation of filopodia. Thus, specific patterns of protein expression and different Eps8-based complexes may be the basis of the different roles that this protein exerts in different experimental systems.

One of the major accomplishments of our study is the demonstration that extracellular cues regulate the subcellular localization and function of Eps8 through phosphorylation. This mode of regulation defines a novel mechanism through which signaling cascades, particularly those emanating from receptor tyrosine kinases, may regulate cappers. Indeed, how the activity of capping proteins is controlled in a signaling-dependent manner has remained a critical, only partially addressed issue. Phosphoinositides, whose generation is controlled through the balance of signaling-regulated lipid kinases and phosphates, can bind and inhibit both gelsolin and CP [58],[59]. Albeit recent evidence obtained using yeast CP questioned whether phosphatidylinositol 3,4-bisphosphate (PIP2) can access CP when it is bound to ends [60], thus casting doubts that this lipid may effectively free barbed ends from capping. Protein:protein interaction is another way to control the activity of capping proteins. In this respect, Carmil, an adaptor protein, was shown to bind CP with high affinity and to inhibit capping, influencing the formation of lamellipodia [61]. Conversely, Abi1 functions as an activator of the otherwise inhibited Eps8.

We show here that BDNF, in particular, controls the barbed-end capping activity of the Eps8:Abi1 complex and modulates Eps8 interaction with the cytoskeleton, strengthening the importance of spatially restricted activation of proteins in the regulation of actin dynamics. Accordingly, BDNF-mediated detachment of Eps8 from actin cytoskeleton correlates with increased filopodia formation, which can be mimicked by genetic removal of this capper. At the molecular level, this is achieved through MAPK-mediated phosphorylation of Eps8 residues S624 and T628, which controls the protein capping activity, with a 10-fold reduction in barbed-end binding of the Eps8:Abi1 complex, following S624 and T628 phosphorylation. Since Eps8 localization depends on both binding to Abi1 [62] and to actin, this reduction in actin binding reasonably contributes to Eps8 subcellular relocalization in response to BDNF. Of note, BDNF-induced detachment of Eps8 from cytoskeletal structures is not accompanied by that of VASP, which remains enriched in the Triton-insoluble fraction, where it may more efficiently associate to filament ends, promoting their elongation.

The regulation of actin-capping activity of Eps8 may have consequences for the developing and adult brain. It is now widely accepted that presynaptic specializations, which are highly dynamic and are continuously added and eliminated throughout the lifetime of a neuron, play an active role in activity-dependent synapse formation and remodeling. In the mature neocortex in vivo, for example, filopodia and short axonal branches are frequently extended and retracted to form boutons onto postsynaptic structures, or to originate en-passant boutons, which are added and eliminated along the axonal shaft (reviewed in [63]). Notably, *eps8* null brains, at early postnatal stages, display a significantly higher number of presynaptic boutons (unpublished data) that might originate from the increased axonal filopodia during neuronal development, thus suggesting a role of the protein in controlling synapse formation in vivo. The dynamic regulation of *eps8* actin-capping activity may therefore play an important role in presynaptic plasticity phenomena in the developing brain and, possibly, in the adult brain.

## Materials and Methods

### Expression Vectors, Reagents, and Antibodies

Cytomegalovirus (CMV) promoter–based and elongation factor-1 (EF1)-promoter–based eukaryotic expression vectors and GST bacterial expression vectors were generated by recombinant PCR.

Polyclonal and monoclonal antibodies (abs) against Eps8 and Abi1 were previously described [42],[64]. Monoclonal abs against synaptobrevin/VAMP2, synaptophysin, SNAP-25, NR1, and synaptotagmin I were from Synaptic System. Monoclonal abs against Gap43, actin, phosphorylated ERK1,2, and beta-tubulin were purchased from Sigma. Monoclonal ab against phosphorylated JNK was from New England Biolab. Polyclonal ab against synapsin I was a kind gift from P. De Camilli (Yale University), ab against riboforin was from G. Kreibich (New York University), and ab against human cathepsin D was from C. Isidoro (Università di Novara). Antibody against fascin was from Dako: clone 55K-2. Antibody against VASP was from BD Transduction Laboratories or from Dr. F. Gertler. GST-VASP was a gift from Dr. C. Le Clainche (CNRS, Gif-sur-Yvette, France). Mouse monoclonal anti-IRSp53 were raised against full-length histidine-tagged recombinantly purified proteins.

FITC-phalloidin and Texas Red phalloidin were purchased from Sigma and Molecular Probes, respectively. The secondary abs conjugated to FITC, Rhodamine, or horse radish peroxidase were obtained from Jackson Immunoresearch Laboratories.

Recombinant human BDNF was obtained from Regeneron. PD98059 is from Sigma.

NSC23766 is a kind gift from Y. Zheng (Cincinnati Children's Hospital Medical Center)

Shrimp Alkaline Phosphatase was from Roche Diagnostics. Monoclonal anti-pY, activated Erk1, and purified MBP were purchased from Upstate.

The Eps8 S624A, T628A (SATA), and S624E, T628E (SETE) mutants were generated by PCR (SATA 1861-TCT GCC CCA (T→ G)CA CCC CCT CCA (A→G)CG CCA GCA CCC-1893; SETE 1861-TCT GCC CCA (TC→GA)A CCC CCT CCA (AC→ GA)G CCA GCA CCC-1893).

### Animal Experiment

The Eps8-KO mice obtained as previously described [42] were backcrossed for more than ten generations to C57BL/6 mice. For preparation of hippocampal culture, littermates derived from heterozygous crosses were used; in some experiments, WT and knockout (KO) mice from F2N12 homozygous colonies were also used. All experiments were performed in accordance with the guidelines established in the IFOM-IEO Campus Principles of Laboratory Animal Care (directive 86/609/EEC).

### Hippocampal Cell Cultures

Primary neuronal cultures were prepared from the hippocampi of 18-d-old fetal rats as described [65] or E16-P1 WT and *eps8* null mice. The dissociated cells were plated onto glass coverslips coated with poly-l-lysine at densities ranging from 10,000 to 20,000 cells/cm^2^. After a few hours, the coverslips were transferred to dishes containing a monolayer of cortical glial cells. The cells were maintained in MEM (Gibco) without sera, supplemented with N2 (Gibco), 2 mM glutamine, and 1 mg/ml BSA (neuronal medium).

### Small RNA Interference

Three different double-strand small interfering RNA (siRNA) oligonucleotides (Stealth RNAi; called 1525, 1526, and 1158) were designed against mouse *eps8* by using RNAi Design Services from Invitrogen. Oligonucleotide sequences:

1525: 5′-GCCATGCCTTTCAAGTCAACTCCTA-3′;

1526: 5′-CCATGCCTTTCAAGTCAACTCCTAA-3′;

1158: 5′-GACAAAAGACACAGTTGATTTCTTAA-3′.

Stealth RNAi negative control with medium content of CG (Invitrogen) whose sequence is not homologous to any vertebrate sequence/transcriptome was used as control. WT mouse hippocampal neurons were transfected with Lipofectamine 2000 (Invitrogen) and with each oligonucleotide sequence at the dose of 200 nM the day of plating, fixed at 3DIV, and stained with anti-VAMP2 abs and phalloidin to evaluate filopodia density.

### Lentiviral Vectors and Lentiviral Infection

GFP alone or GFP-eps8 WT or eps8MUT1 were subcloned in a pRRLsin.PPT.CMV lentiviral vector (kind gift of L. Naldini, San Raffaele, Milano). The 293T human embryonic kidney cells were cotransfected with the lentiviral vector and the packaging plasmids (pMDL, pRev, and pVSVG), providing the required *trans*-acting factors, namely, Gag-Pol, Rev, and the envelope protein VSVG, respectively, and viruses were obtained as described [66]. Primary cultures established from *eps8^−/−^* mice were infected 1 d after plating with GFP/GFP-Eps8/GFP-Eps8-MUT1 lentiviruses. Medium was changed after 6 h of incubation, and cells were fixed 48 h after viral infection.

### Immunocytochemistry

The cultures were fixed and stained as previously described [38]. The images were acquired using a BioRad MRC-1024 confocal microscope equipped with LaserSharp 3.2 software.

### Image Analysis and Statistics

Acquired images were analyzed using Metamorph Imaging Series 6.1 software (Universal Imaging). The number of filopodia and the length of axonal shaft were then computed in order to obtain the filopodia density (number of filopodia/micrometer). For each neuron, an axon length ranging between 30 and 80 µm was analyzed. Alternatively, the percentage of neurons with filopodia was evaluated. In this latter case, neurons with more than 0.04 filopodia/µm were considered as filopodia-bearing neurons. For each experimental condition, both types of analyses gave similar results.

Statistical analysis was performed using SigmaStat 2.0 software (Jandel Scientific). After testing whether data were normally distributed or not, the appropriate statistical test has been used. Detailed information is reported in the figure legends. Statistical significance is indicated in graphs as follows: a single asterisk (*) indicates *p*<0.05; double asterisks (**), *p*<0.001.

### Biochemical Techniques

#### Growth cone particles

GCPs were prepared as described [35],[36]. Isolated GCPs were diluted in seven to eight volumes of modified Krebs Ringer solution (180 mM sucrose, 50 mM NaCl_2_, 5 mM KCl, 22 mM Hepes (pH 7.4), 10 mM glucose, 1.2 mM NaH_2_PO_4_, 1.2 mM MgCl) containing, if needed, an antagonist (PD98059 30 µM) and kept on ice for 5 min. For stimulation, GCPs were transferred to 37°C for 5–10 min, then an agonist (BDNF 100 ng/ml) was added for 20 min. A fraction of GCP was immediately resuspended in SDS containing sample buffer for analysis of phosphoproteins. The remaining sample was extracted in 1% Triton X-100 for 10 min on ice and subsequently centrifuged for 1 h at 100,000*g*. Both pellet (TX-100–insoluble fraction) and supernatant (TX-100–soluble fraction) were processed by SDS gel electrophoresis followed by western blotting.

#### Synaptosome preparation

The purification of synaptosomes from rat forebrain was carried out as described [67]. Samples with a protein content of 200 µg were pelleted and resuspended in KRH devoid of Ca^2+^, but containing PD98059 if needed. The samples were kept for 10 min on ice and then transferred for 10 min to 37°C. After addition of BDNF, they were left for 10 min at 37°C, then a fraction corresponding to 50 µg of protein was mixed with SDS containing sample buffer for phosphoprotein analysis. The remaining sample was processed for two-dimensional gel electrophoresis (2DE).

#### Two-dimensional gel electrophoresis

Immobilized pH gradient (IPG) gel strips (pH 3–10 nonlinear [NL], 7 cm; General Electric Healthcare) were used as the first-dimension gel of 2DE for isoelectric focusing. GCPs (ca. 250 µg) or synaptosomes (125 µg) were solubilized overnight on a rotating wheel at 4°C by adding 6.4 volumes of distilled water with 1% TritonW-100 f.c.(final concentration) and protease inhibitor cocktail (Sigma; 1∶1,000 f.c.), Phosphatase Inhibitor Cocktail I and II (Sigma; 1∶1,000 f.c.) and desoxyribonuclease 10 µg/ml. Proteins were then precipitated with cold acetone to a final concentration of 80%. The pellet obtained was resuspended in 125 µl of rehydration buffer containing 7 M urea, 2 M thiourea, 2% Triton, 2% chaps, 1% IPG buffer ampholyte (pH 3–10 NL; General Electric Healthcare), 50 mM DTT, and a trace amount of bromophenol blue; gels rehydrated and subjected to isoelectric focusing at 18°C with a stepwise-increased voltage (rehydration 0 V for 1 h; 30 V for 12 h; 500 V for 1 h; 1,000 V for 1.5 h; 5,000 V for 24,000 V×h). After the first dimensional electrophoresis, the strips were incubated in equilibration buffer (50 mM Tris-HCl [pH 6.8], 6 M urea, 30% glycerol, 2% SDS) with 20 mg/ml DTT for 20 min, followed by 20 min in equilibration buffer with 25 mg/ml iodoacetamide, and resolved in 8% SDS-polyacrylamide mini gels as second dimensional electrophoresis.

#### Protein dephosphorylation with alkaline phosphatase

Dephosphorylation of Eps8 was investigated by treatment with alkaline phosphatase (AP). For this experiment, BDNF-treated synaptosomes (ca 125 µg) were solubilized in adequate volume of dephosphorylation buffer (1% Triton-X100, 50 mM TRIS [pH 8.0], 1 mM MgCl_2_, 1 mM EDTA) with protease inhibitor cocktail (1∶1,000 f.c.) and desoxyribonuclease 10 µg/ml on a rotating wheel at 4°C overnight. Then synaptosomes were centrifuged for 30 min at 16,000 rpm in a refrigerated microcentrifuge (Eppendorf). The supernatant was treated with 20 units of AP for 1 h at 37°C. After, samples were processed as usual for 2DE by adding 6.4 volumes of distilled water with 1% TritonW-100 f.c. and protease inhibitor cocktail (Sigma, 1∶1,000 f.c.), and desoxyribonuclease 10 µg/ml, and proteins were precipitated with four volumes of cold acetone.

#### Cell fractionation

HeLa cells were transfected with the indicated expression vectors using Lipofectamine (Invitrogen), according to the manufacturer's instructions. One day after transfection, cells were collected and counted. An equal number of cells were lysed in TX buffer (15 mM Hepes [pH 7.2], 80 mM NaCl, 2 mM MgCl_2_, 0.1% Triton X-100, protease inhibitor cocktail [Roche]). After 10 min on ice, cells lysates were subjected to ultracentrifugation (145,000*g*, 45 min, 4°C). Supernatants corresponded to the Triton X-100–soluble, cytosolic-enriched fractions; pellets were solubilized in equal volumes of RIPA buffer (15 mM Hepes [pH 7.2], 140 mM NaCl, 3 mM MgCl_2_, 1 mM EDTA, 0.5% sodium deoxycholate, 1% NP-40, 2% SDS, protease inhibitor cocktail [Roche]), subjected to extensive sonication, and centrifuged at 16,000*g* for 20 min at 4°C. Supernatants represented the Triton X-100–insoluble, membrane- and F-actin–enriched fractions. Equal amounts of each fraction were loaded, resolved on SDS-PAGE, and immunoblotted with the appropriate antibodies. Biochemical fractionation experiments were performed at least three times unless otherwise indicated.

#### Eps8 purification

His-Eps8 FL WT, SATA, or SETE were purified from human 293T cells. Briefly, 293T cells transfected with pCDNA-HisMax4-Eps8s were lysed in 20 mM phosphate buffer (pH 8.0), 500 mM NaCl, 20 mM imidazole, 1 mM β-mercapto-ethanol, 5% glycerol, protease inhibitor cocktail (Roche) and sonicated. His-Eps8s were purified using Ni-NTA-agarose (Qiagen) following standard procedures. Recombinant Eps8 fragments were expressed as GST fusion proteins in the BL21 *Escherichia coli* strain (Stratagene), and affinity purified using GS4B glutathione–sepharose beads (Amersham Pharmacia Biotech). Eluted proteins were dialysed in 50 mM Tris-HCl, 150 mM NaCl, 1 mM DTT, and 20% glycerol.

#### In vitro phosphorylation assay

Purified His-Eps8, Eps8 GST-fragments or MBP (Upstate) were incubated with 10 µM [γ-32P]-ATP (Amersham Pharmacia Biotech) in kinase buffer (20 mM Tris HCl [pH 7.5], 10 µM orthovanadate, 10 mM MgCl_2_, 50 mM NaCl, 1 mM DTT, 1 mM NaF) in the presence or absence of purified MAP Kinase 1/Erk1, active (Upstate) for 30 min at 30°C. Phosphorylation of the Eps8 GST fragments used in actin polymerization assays was as follow: GST Eps8 535–821 was incubated in the presence of an excess of cold ATP (10 µM) with or without MAPK.

#### Actin polymerization assays

Actin cosedimentation assays were performed as described [28]. Briefly, monomeric rabbit G-actin was induced to polymerize at 30°C in F-actin buffer (5 mM Tris-HCl [pH 7.8], 0.2 mM ATP, 1 mM DTT, 0.1 mM CaCl_2_, 1 mM MgCl_2_, and 100 mM KCl). Recombinant and purified GST-fusion proteins or control buffer were subsequently incubated with F-actin and ultracentrifuged for 30 min at 400,000*g* in a Beckman TL-100 table-top ultracentrifuge. Equal amounts of starting materials, supernatants, and pellets were solubilized in loading buffer, boiled, and then resolved on an SDS–PAGE gel together with standards of actin and Eps8 proteins.

Actin polymerization was monitored by the increase in fluorescence of 10% pyrenyl-labeled actin. Seeded polymerization was induced by addition of 0.1 M KCl, 1 mM MgCl_2_ and 0.2 mM EGTA to a solution of Ca-ATP–G-actin (2.5 µM) containing spectrin–actin seeds (0.25 nM) and the desired amount of recombinant proteins. Fluorescence measurements were performed at 20°C in a Safas Sfx or a Spex Fluorolog 2 spectrofluorimeter. The initial rate of barbed-end growth was measured. The affinity for barbed ends was derived as described in [28].

Actin bundling assays were performed as described in [27].

#### Crib assays for Rac activation

The levels of Rac-GTP were measured by affinity precipitation using GST-CRIB (Cdc42 and Rac interactive region) of PAK1, as described in [42]. In brief, cells or tissues were lysed in buffer containing 50 mM Tris-HCl (pH 7.5), 150 mM NaCl, 10 mM MgCl_2_, 1% NP-40, 5% glycerol, and 1 mM EDTA (pH 8), 1 mM PMSF, and Protease Inhibitor Cocktail Set III (Calbiochem). Lysates were incubated with 30 µg of GST-CRIB protein immobilized on glutathione-Sepharose (AmershamBiosciences) beads, for 1 h at 4°C. Specifically bound GTP-Rac was detected by immunoblot with anti-Rac1.

## Supporting Information

Figure S1 **Functional interference of VASP significantly reduces the number of axonal filopodia in** ***eps8*** **null neurons.** Left panel, 3DIV *eps8^−/−^* hippocampal neurons expressing FPPPmitoGFP [17] or GFP as control (green) were stained with anti-VASP antibody (blue) and Texas Red phalloidin (red). Expression of FPPPmitoGFP, which displaces VASP from its normal sites of localization, sequestering it to the mitochondrial surface, heavily decreases the number of filopodia protruding from the axon (right graph, Mann-Whitney rank sum test, *p*<0.001). Arrowhead points to a not-transfected process bearing filopodia. Bar indicates 6 µm for (E).
(5.13 MB EPS)

Figure S2 **Down-regulation of Eps8 by siRNA or genetic approaches promotes the formation of neuronal filopodia.** (A–F) Down-regulation of Eps8 by siRNA effectively reduced protein levels in NIH3T3 cells and hippocampal neurons, where it leads to increased filopodia density. (A and B) Lysates of NIH3T3 cells transfected with three different siRNA oligonucleotides against Eps8 (1525, 1226, and 1158) or a scramble oligo (CTR) were immunoblotted with anti-Eps8 or anti-tubulin, as a loading control, antibodies. (B and C) The efficacy of the oligonucleotide 1525 to down-regulate Eps8 expression is shown in NIH3T3 cells (B) and primary hippocampal neurons (C) by immunofluorescence with an anti-Eps8 antibody. Similar results were obtained using the other two oligos (unpublished data). Asterisks in (B) indicate the nucleus of RNAi oligo-transfected cells. Bars indicate 10 µm. (D) Examples of axonal shafts from scrambled, control (CTR)- and 1525-transfected neurons stained for synaptobrevin/VAMP2. Bar indicates 6 µm. (E) Quantification of the density of filopodia of cultured hippocampal neurons (3DIV), untransfected (unt) or transfected with a scrambled oligonucleotide (scrbld), or with the three oligonucleotides against Eps8. (Kruskal-Wallis one-way analysis of variance [ANOVA] on ranks, *p*≤0.001). Data are expressed as the mean±SE. (F and G) Genetic removal of *eps8* causes increased number of dendritic filopodia in hippocampal neurons. (G) WT and *eps8^−/−^* hippocampal neurons were transfected with EGFP and examined at 7DIV. Dendritic filopodia are visualized by EGFP fluorescence. (H) Quantification of the density of dendritic filopodia in WT and *eps8* null hippocampal neurons (Mann-Whitney rank sum test, *p*<0.001). Bars indicate 12 µm for low-magnification images, and 8 µm for high-magnification images.
(6.23 MB TIF)

Figure S3 **Biochemical characterization of Eps8MUT1.** (A) Eps8MUT1 lacks barbed-end capping. Kinetics of actin polymerization induced by spectrin–actin seeds in the presence of GST-Eps8 648–821 (250 nM) or GST-Eps8 648-821 MUT1 (250 nM) bearing three point mutations (V729A, T731A, and W733A). Fluorescence was monitored over time. Whereas Eps8-648-821 completely inhibits actin polymerization at substoichiometric concentrations with a constant of dissociation for barbed ends of *K*_d_ = 4 nM (unpublished data), as previously reported [28], Eps8-648-821 MUT1 displays no significant barbed-end capping activity, as indicated by its constant of dissociation for barbed ends, which is 1,000-fold higher thaN Eps8 648-821 (*K*_d_ = 4,000 nM, unpublished data), while retaining filament binding and cross-linking activity (see below). (B). Eps8MUT1 binds to actin filaments, such as Eps8, in ultracentrifugation assays. GST-Eps8 648–821 or GST-Eps8 648–821 MUT1 (GST-MUT1) were incubated either alone (−) or in the presence (+) of the indicated concentrations of F-actin and subjected to ultracentrifugation. Pellets and supernatants were immunoblotted with the indicated abs. The amounts of F-actin in pellet and supernatant in absence of GST fusion proteins were also determined. (C) Eps8MUT1 cross-links actin filaments. Filamentous actin (1 µM) was incubated either in the presence of GST (left panel) or the indicated concentration of GST-Eps8 648–821 or GST-Eps8 648–821 MUT1 (GST-MUT1). The reaction mixture was incubated at room temperature for 30 min. Actin was then labeled with rhodamine-phalloidin, and DABCO 0.1% and methyl cellulose 0.1% were added to the mixture. The samples were mounted between a slide and a coverslip coated with poly-lysine and imaged by fluorescence microscopy. Bar indicates 2 µm. The rationale to engineer the three mutations (V729A, T731A, and W733A) stems from the following set of observations: we found that the eps8 capping domains display sequence, secondary, and tertiary structure similarity with the SAM Pointed (SAM_PNT) domain, which belong to the larger family of sterile alpha motif (SAM) [68]. The recently solved nuclear magnetic resonance (NMR) structure of the C-terminal region of human EPS8L2 (residues 612–697, 1WWU), a member of the EPS8L family protein [25], and of human EPS8 (residues 699–784, 2E8M) deposited in the Protein Data Bank (PDB) database, fully confirmed this assertion. This domain is composed of five consecutive alpha helices, from N- to C-terminus, named alpha1 to alpha5. We previously reported through structure-function analysis using various Eps8 fragments that the one encompassing amino acids (aa) 674–737 and including the two first alpha helices is sufficient to mediate actin capping [28], but loses actin bundling [27], indicating that the integrity of these helices is critical for capping. We thus mutated three residues predicted on the basis of secondary structures and the NMR data to disrupt zand thus to hamper capping, but not bundling. We verified these predictions experimentally as described above. A detailed structural and biochemical analysis of Eps8 mode of capping actin filaments is currently on-going and will be described elsewhere.
(3.76 MB EPS)

Figure S4 **Contribution of Abi1 and Abi2 (A), rac activation (B–E), and tyrosine phosphorylation (F) to Eps8 function.** (A) Abi1 and Abi2 coimmunoprecipitate with Eps8 in brain lysates, but not in HeLa cells. Left panels: total lysates from either brain cortex and hippocampi (3 mg) (H/C) or HeLa cells were immunoprecipitated as indicated on top. Lysates (50 µg) and immunoprecipitates were analyzed by immunoblotting with the indicated antibodies. Right panel: equal amounts of cell lysates from HeLa cells or mouse primary hippocampal neurons (Hip) were immunoblotted with the indicated antibodies to analyze the relative expression levels of Eps8 and its binding partners (Abi1, Abi2, and IRSp53)**.** The different electrophoretic pattern of IRSp53 between HeLa and hippocampi reflects the existence of multiple splice variants differentially expressed in different tissues [69]–[71]. (B–E) Eps8 removal causes increased filopodia in a Rac-independent manner. Eps8 removal does not impair RacGTP levels in brain. (B) The levels of RacGTP in brain cortex and hippocampus derived from WT and *eps8^−/−^* mice are similar. Equal amounts of total tissue protein extract from brains of adult wild-type (WT) or *eps8^−/−^* (KO) littermates were incubated in the presence of GST-fused Pak1-CRIB (Cdc42 and Rac interactive binding motif). Lysates from total tissue protein extract (lower panel, Rac) and GST-CRIB (upper panel, RacGTP) bound proteins were immunoblotted with anti-Rac antibodies. (C and D) Efficacy of the Rac inhibitor NSC23766 in mouse embryo fibroblasts and in hippocampal neurons. (C) Serum-starved NIH3T3 cells were incubated with NSC23766 (50 µM) or vehicle (mock) for 48 h before addition of PDGF(100 ng/ml for 7 min) or mock treatment. Cells were then fixed and labeled with FITC-phalloidin. The Rac1 inhibitor NSC23766 completely abrogates actin ruffles induced by PDGF. (D) Levels of RacGTP in hippocampal cultures (3DIV) upon exposure to 50 µM NSC23766 for 3 h determined by affinity CRIB-based assay as described in (B). (E) Quantification of filopodia density in hippocampal neurons (3DIV) treated with NSC23766 (50 µM for 3 h); the histogram shows the normalized mean number of axonal filopodia per micronmeter (Mann-Whitney rank sum test, *p*≤0.001). Data are the mean±SE. Bar indicates 20 µm. (F) BDNF stimulation does not induce Eps8 tyrosine phosphorylation. Pellets from rat forebrain synaptosomes preparations (1 mg, see Materials and Methods) mock-treated (−) or treated with BDNF (+) 100 ng/ml 20 min, where resuspended in 50 mM HEPES (pH 7.5), 1% glycerol, 50 mM NaCl, 1% Triton X-100, 1.5 mM MgCl_2_, 5 mM EGTA, protease inhibitor cocktail (Sigma, 1∶1000 f.c.), phosphatase inhibitor cocktail I and II (Sigma, 1∶1000 f.c.) at 4°C and subjected to immunoprecipitation with anti-Eps8 (Eps8) or control antibody (CTR). Immunoprecipitates (IPs) and control lysates (Inputs) were resolved by SDS-gel electrophoresis and immunoblotted (IB) with the indicated antibodies.
(4.25 MB TIF)

Figure S5 **Biochemical characterization of Eps8 phosphomutants.** (A) Eps8-SATA is not phosphorylated by MAPK. Recombinant purified His-Eps8 WT or His-Eps8-SATA (3 µg) were incubated with 10 µCi [γ-32P]-ATP in the presence (+) or absence (−) of purified MAPK (20 ng) in kinase buffer (see Materials and Methods). MBP (myelin basic protein) was used as positive control of the reaction. Products were analyzed by SDS-PAGE followed by autoradiography (right panels). The red arrow indicates the shift of Eps8 due to phosphorylation. Left panel shows Coumassie Blue staining analysis of recombinant MBP and Eps8. MAPK-mediated phosphorylation does not affect the barbed-end capping activity of the isolated C-terminal Eps8 535–821 fragment. (B and C) Actin polymerization assay of WT or mutated Eps8 capping domain in the presence of MAPK. (B) Either 5 or 100 nM of Eps8 C-terminal fragment (535–821) phosphorylated by MAPK (P-535) in the presence of an excess of ATP (see Materials and Methods) or Eps8 C-terminal fragments preincubated with only MAPK (535+M) or ATP (535+A) as controls, were mixed to MgATP-G-actin (2.5 µM, 10% pyrenyl-labeled) and 0.25 nM of spectrin–actin seeds. Fluorescence was monitored over time. (C) Phosphomutants of Eps8 (535–821) display similar affinity to barbed ends as Eps8 WT. Increasing amounts of purified Eps8 WT, SATA, or SETE mutant C-terminal regions (535–821 WT, 535–821 SATA, and 535–821 SETE) were mixed to MgATP–G-actin (2.5 µM, 10% pyrenyl-labeled) and 0.25 nM of spectrin–actin seeds. Fluorescence was monitored over time. The relative inhibition of polymerization rate, calculated as described in the Materials and Methods section, was plotted over different concentrations of Eps8 fragments. The affinity for actin barbed ends of each of the Eps8 C-terminal fragments is shown. (D) MAPK-mediated phosphorylation does not affect the binding of Eps8 (535–821) to F-actin. Eps8 C-terminal fragment (GST-535-821) (1 µM) incubated with MAPK in the presence or absence of an excess of ATP (see Materials and Methods) were mixed with 5 µM of F-actin and subjected to cosedimentation assay by high-speed ultracentrifugation. Each lane shows the supernatant (S) and the pellet (P) after ultracentrifugation. (E) Phosphomutants of Eps8 (535–821) display similar binding ability to F-actin as Eps8 WT. Equal molar amounts (1 µM) of Eps8 WT, or SATA or SETE C-terminal fragments (GST-535-821) were incubated either alone or with 5 µM (upper panel) or 2 µM (lower panel) of F-actin and subjected to cosedimentation assay by high-speed ultracentrifugation. Each lane shows the supernatant (S) and the pellet (P) after ultracentrifugation.
(4.27 MB TIF)

Video S1 **The dynamic formation of axonal filopodia is significantly increased in** ***eps8^−/−^*** **as compared to WT hippocampal neurons.** Hippocampal neurons (3DIV) were obtained from E18 WT (top) and *eps8* null (bottom) mice. The dissociated cells were plated onto glass coverslips coated with poly-l-lysine at densities ranging from 10,000 to 20,000 cells/cm^2^. After a few hours, the coverslips were transferred to dishes containing a monolayer of cortical glial cells. Live imaging recording was performed by Zeiss LSM 510 Meta. Frames were taken every 5 s for a total of 4 min. Bar indicates 10 µm. Insets show a magnified detail of filopodia dynamically emerging and retracting from the axonal shaft. It is evident from these movies that the average length of filopodia (3.6±0.8 standard error of the mean [SEM], *n* = 50) is not significantly affected by removal of Eps8, which instead causes an increase in the number of cell-forming filopodia and in the density of filopodia along the axon. This suggests that the capping activity of Eps8 likely regulates initiation rather than elongation of filopodia. Additionally, expression of high levels of ectopic *eps8* blocks the formation of filopodia rather than preventing their elongation (Figure 3).
(2.77 MB MOV)

## Abbreviations

DIV: days in vitro
GCP: growth cone particle
RNAi: RNA interference
WT: wild-type
