# Correction: Eps8 Regulates Axonal Filopodia in Hippocampal Neurons in Response to Brain-Derived Neurotrophic Factor (BDNF)

**DOI:** 10.1371/journal.pbio.1002184

**Published:** 2015-06-03

**Authors:** Elisabetta Menna, Andrea Disanza, Cinzia Cagnoli, Ursula Schenk, Giuliana Gelsomino, Emanuela Frittoli, Maud Hertzog, Nina Offenhauser, Corinna Sawallisch, Hans-Jürgen Kreienkamp, Frank B. Gertler, Pier Paolo Di Fiore, Giorgio Scita, Michela Matteoli

The authors have realized that the composition of the gel image in S5D Fig is not clearly explained in the figure legend, nor is it marked adequately in the figure itself. We provide therefore an amended legend and figure, which clarify the compilation of the S5D Fig and the presentation of the data.

In addition, and at the editors’ request, the original data related to Fig. 6D in this paper were made available. Following verification of these original data, the editors were satisfied with the data provided. A supplementary S6 Fig, which comprises the original film of the immunoblot (upper panel of Fig. 6D) and the original picture of the entire nitrocellulose membrane (lower panel of Fig. 6D) used to reveal GST fusion proteins employed in the pull down experiment described in Fig. 6D, has been included as supplementary material.

## Supporting Information

S5 Fig (D) MAPK-mediated phosphorylation does not affect the binding of Eps8 (535–821) to F-actin. Eps8 C-terminal fragment (GST-535-821) (1 μM) incubated with MAPK in the presence or absence of an excess of ATP were mixed with 5 μM of F-actin and subjected to co-sedimentation assay by high-speed ultracentrifugation. Each lane shows the supernatant (S) and the pellet (P) after ultracentrifugation. The panels show a composite of two gels. All conditions but +ATP/+MAPK were run in one gel, while the sample marked +ATP/+MAPK, including its molecular weight marker (M), were run in a different gel.
(PDF)

S6 Fig (related to Fig. 6D), primary data. Eps8 phosphomutants bind Abi1 similar to Eps8 WT. Lysates of cells expressing GFP-Eps8 WT or SATA or SETE (indicated at the top) were incubated with immobilized GST, or GST-PIN1, used as negative control, or GST-Abi1 as described in Fig. 6D. Lysates and bound proteins were immunoblotted with anti-GFP antibody (upper panel) or stained to reveal GST (lower panels). The full scan of the original immunoblot and of the picture of the entire nitrocellulose membrane are shown. Mw markers are indicated on the left. Boxed regions indicate the cropped blot used to assemble Fig. 6D.
(TIF)
