# Supplementary figures and images for: Eps8 Regulates Axonal Filopodia in Hippocampal Neurons in Response to Brain-Derived Neurotrophic Factor (BDNF)
Source: PLoS Biol. 2009 Jun 30;7(6):e1000138. doi: 10.1371/journal.pbio.1000138 (PMC2696597; doi:10.1371/journal.pbio.1000138)

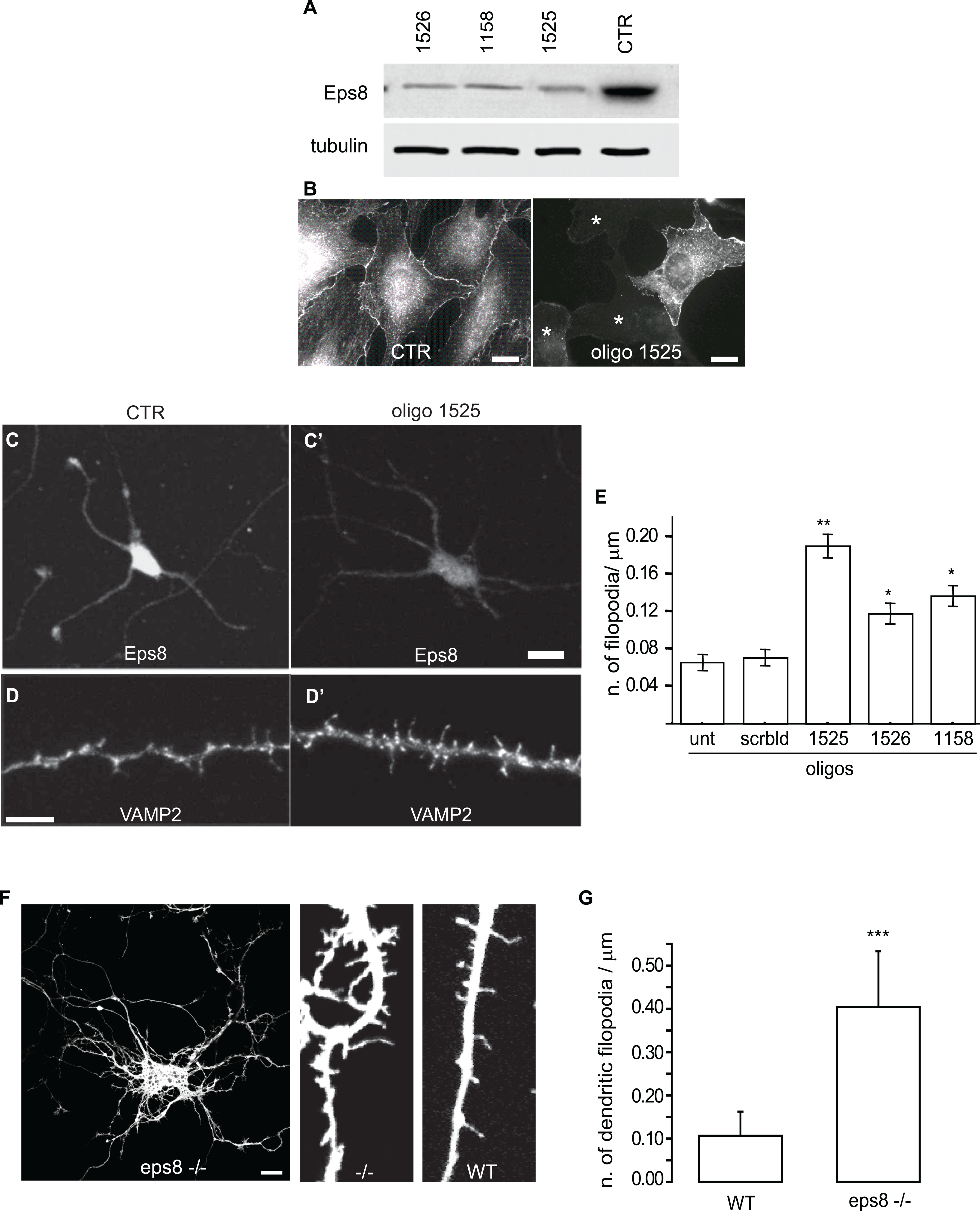

Supplement: Figure S2 — Down-regulation of Eps8 by siRNA or genetic approaches promotes the formation of neuronal filopodia. (A–F) Down-regulation of Eps8 by siRNA effectively reduced protein levels in NIH3T3 cells and hippocampal neurons, where it leads to increased filopodia density. (A and B) Lysates of NIH3T3 cells transfected with three different siRNA oligonucleotides against Eps8 (1525, 1226, and 1158) or a scramble oligo (CTR) were immunoblotted with anti-Eps8 or anti-tubulin, as a loading control, antibodies. (B and C) The efficacy of the oligonucleotide 1525 to down-regulate Eps8 expression is shown in NIH3T3 cells (B) and primary hippocampal neurons (C) by immunofluorescence with an anti-Eps8 antibody. Similar results were obtained using the other two oligos (unpublished data). Asterisks in (B) indicate the nucleus of RNAi oligo-transfected cells. Bars indicate 10 µm. (D) Examples of axonal shafts from scrambled, control (CTR)- and 1525-transfected neurons stained for synaptobrevin/VAMP2. Bar indicates 6 µm. (E) Quantification of the density of filopodia of cultured hippocampal neurons (3DIV), untransfected (unt) or transfected with a scrambled oligonucleotide (scrbld), or with the three oligonucleotides against Eps8. (Kruskal-Wallis one-way analysis of variance [ANOVA] on ranks, p≤0.001). Data are expressed as the mean±SE. (F and G) Genetic removal of eps8 causes increased number of dendritic filopodia in hippocampal neurons. (G) WT and eps8−/− hippocampal neurons were transfected with EGFP and examined at 7DIV. Dendritic filopodia are visualized by EGFP fluorescence. (H) Quantification of the density of dendritic filopodia in WT and eps8 null hippocampal neurons (Mann-Whitney rank sum test, p<0.001). Bars indicate 12 µm for low-magnification images, and 8 µm for high-magnification images. (6.23 MB TIF) [file pbio.1000138.s002.tif]

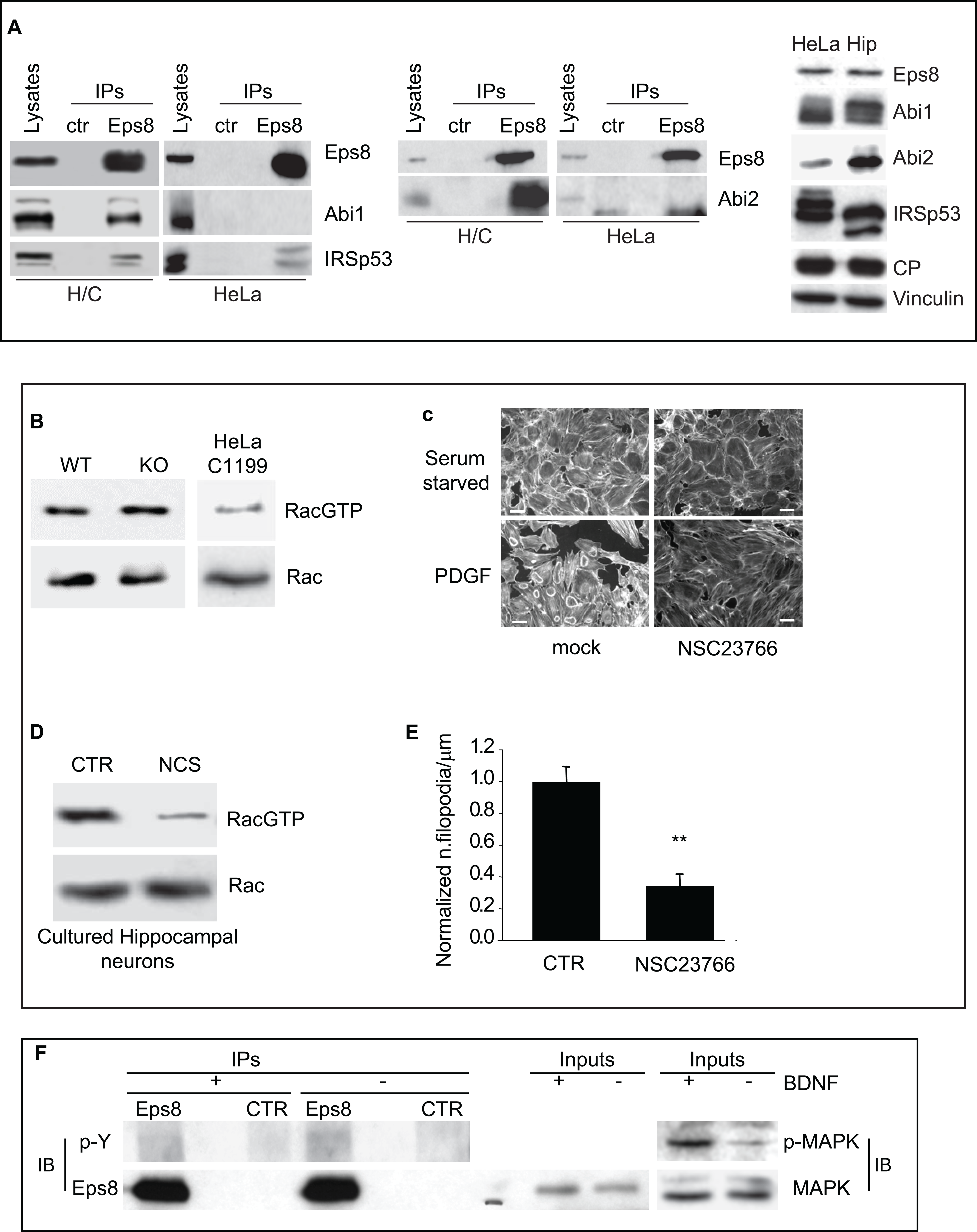

Supplement: Figure S4 — Contribution of Abi1 and Abi2 (A), rac activation (B–E), and tyrosine phosphorylation (F) to Eps8 function. (A) Abi1 and Abi2 coimmunoprecipitate with Eps8 in brain lysates, but not in HeLa cells. Left panels: total lysates from either brain cortex and hippocampi (3 mg) (H/C) or HeLa cells were immunoprecipitated as indicated on top. Lysates (50 µg) and immunoprecipitates were analyzed by immunoblotting with the indicated antibodies. Right panel: equal amounts of cell lysates from HeLa cells or mouse primary hippocampal neurons (Hip) were immunoblotted with the indicated antibodies to analyze the relative expression levels of Eps8 and its binding partners (Abi1, Abi2, and IRSp53). The different electrophoretic pattern of IRSp53 between HeLa and hippocampi reflects the existence of multiple splice variants differentially expressed in different tissues [69]–[71]. (B–E) Eps8 removal causes increased filopodia in a Rac-independent manner. Eps8 removal does not impair RacGTP levels in brain. (B) The levels of RacGTP in brain cortex and hippocampus derived from WT and eps8−/− mice are similar. Equal amounts of total tissue protein extract from brains of adult wild-type (WT) or eps8−/− (KO) littermates were incubated in the presence of GST-fused Pak1-CRIB (Cdc42 and Rac interactive binding motif). Lysates from total tissue protein extract (lower panel, Rac) and GST-CRIB (upper panel, RacGTP) bound proteins were immunoblotted with anti-Rac antibodies. (C and D) Efficacy of the Rac inhibitor NSC23766 in mouse embryo fibroblasts and in hippocampal neurons. (C) Serum-starved NIH3T3 cells were incubated with NSC23766 (50 µM) or vehicle (mock) for 48 h before addition of PDGF(100 ng/ml for 7 min) or mock treatment. Cells were then fixed and labeled with FITC-phalloidin. The Rac1 inhibitor NSC23766 completely abrogates actin ruffles induced by PDGF. (D) Levels of RacGTP in hippocampal cultures (3DIV) upon exposure to 50 µM NSC23766 for 3 h determined by affinity CRIB-based assay [file pbio.1000138.s004.tif]

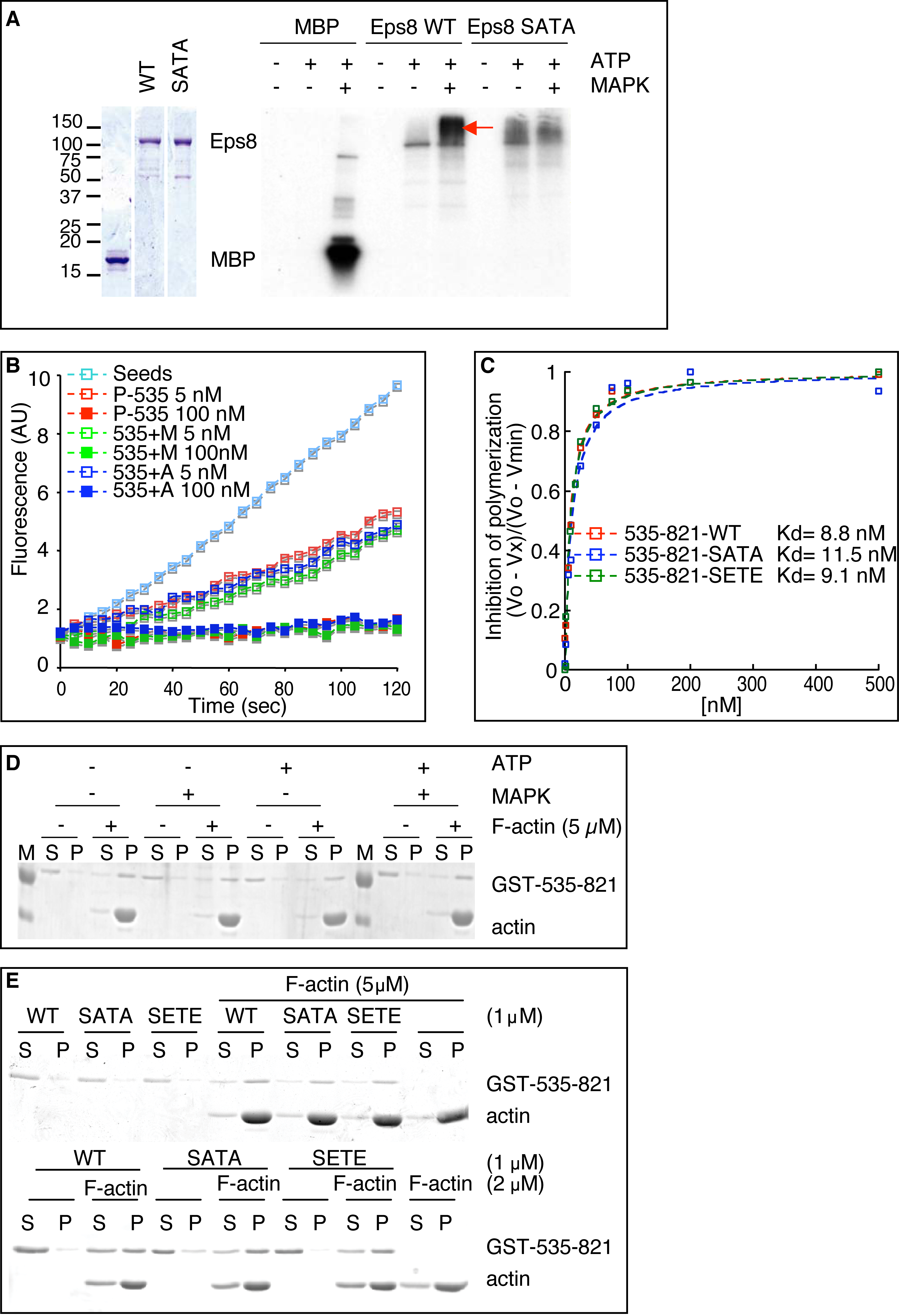

Supplement: Figure S5 — Biochemical characterization of Eps8 phosphomutants. (A) Eps8-SATA is not phosphorylated by MAPK. Recombinant purified His-Eps8 WT or His-Eps8-SATA (3 µg) were incubated with 10 µCi [γ-32P]-ATP in the presence (+) or absence (−) of purified MAPK (20 ng) in kinase buffer (see Materials and Methods). MBP (myelin basic protein) was used as positive control of the reaction. Products were analyzed by SDS-PAGE followed by autoradiography (right panels). The red arrow indicates the shift of Eps8 due to phosphorylation. Left panel shows Coumassie Blue staining analysis of recombinant MBP and Eps8. MAPK-mediated phosphorylation does not affect the barbed-end capping activity of the isolated C-terminal Eps8 535–821 fragment. (B and C) Actin polymerization assay of WT or mutated Eps8 capping domain in the presence of MAPK. (B) Either 5 or 100 nM of Eps8 C-terminal fragment (535–821) phosphorylated by MAPK (P-535) in the presence of an excess of ATP (see Materials and Methods) or Eps8 C-terminal fragments preincubated with only MAPK (535+M) or ATP (535+A) as controls, were mixed to MgATP-G-actin (2.5 µM, 10% pyrenyl-labeled) and 0.25 nM of spectrin–actin seeds. Fluorescence was monitored over time. (C) Phosphomutants of Eps8 (535–821) display similar affinity to barbed ends as Eps8 WT. Increasing amounts of purified Eps8 WT, SATA, or SETE mutant C-terminal regions (535–821 WT, 535–821 SATA, and 535–821 SETE) were mixed to MgATP–G-actin (2.5 µM, 10% pyrenyl-labeled) and 0.25 nM of spectrin–actin seeds. Fluorescence was monitored over time. The relative inhibition of polymerization rate, calculated as described in the Materials and Methods section, was plotted over different concentrations of Eps8 fragments. The affinity for actin barbed ends of each of the Eps8 C-terminal fragments is shown. (D) MAPK-mediated phosphorylation does not affect the binding of Eps8 (535–821) to F-actin. Eps8 C-terminal fragment (GST-535-821) (1 µM) incubated with MAPK in the presence or absence of an [file pbio.1000138.s005.tif]
