# Supplementary figures and images for: Correction: Eps8 Regulates Axonal Filopodia in Hippocampal Neurons in Response to Brain-Derived Neurotrophic Factor (BDNF)
Source: PLoS Biol. 2015 Jun 3;13(6):e1002184. doi: 10.1371/journal.pbio.1002184 (PMC4454587; doi:10.1371/journal.pbio.1002184)

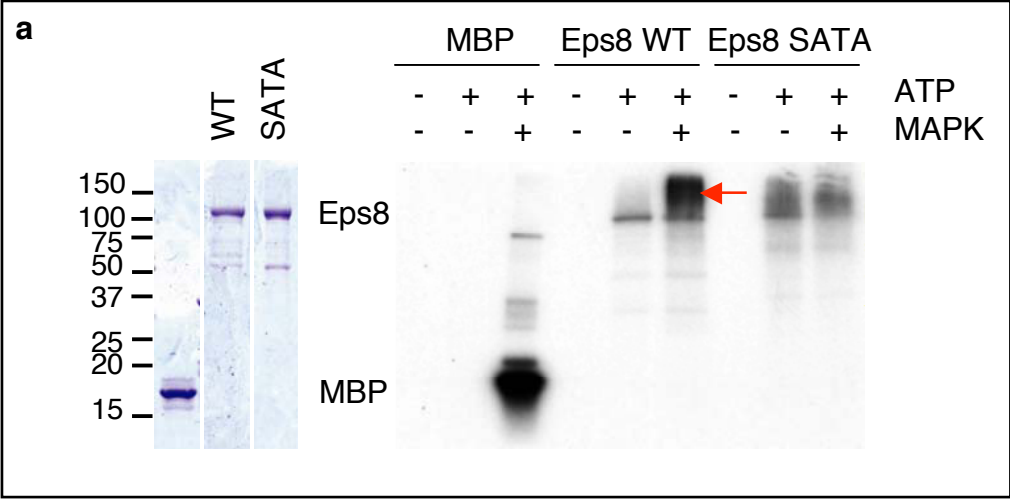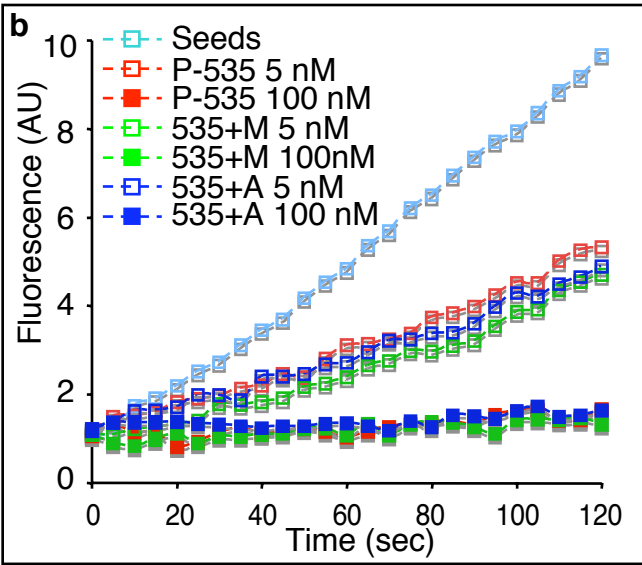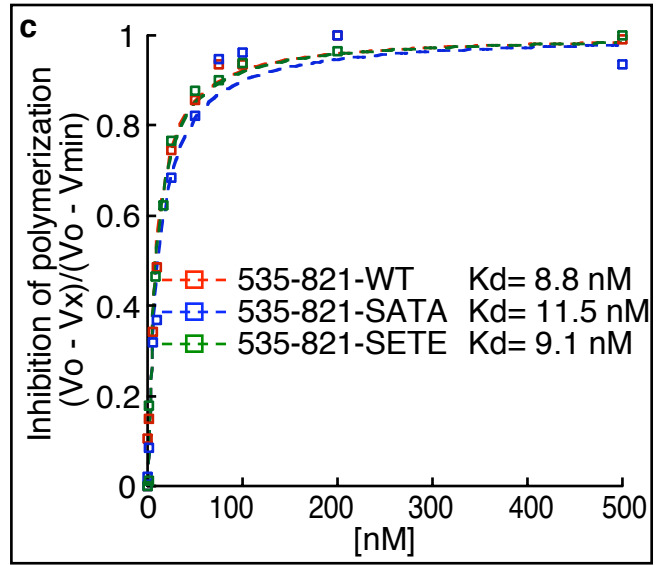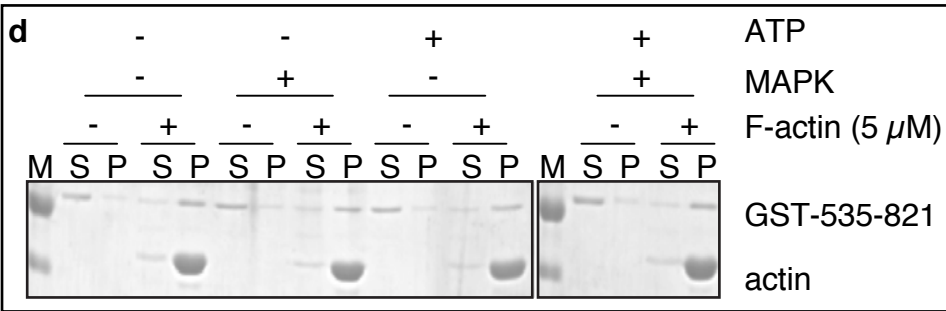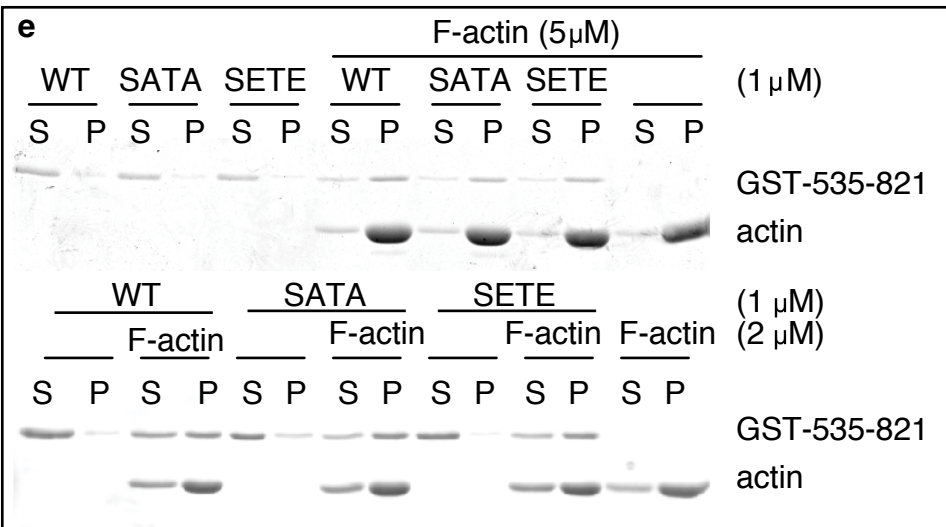

Supplement: S5 Fig — Eps8 C-terminal fragment (GST-535-821) (1 μM) incubated with MAPK in the presence or absence of an excess of ATP were mixed with 5 μM of F-actin and subjected to co-sedimentation assay by high-speed ultracentrifugation. Each lane shows the supernatant (S) and the pellet (P) after ultracentrifugation. The panels show a composite of two gels. All conditions but +ATP/+MAPK were run in one gel, while the sample marked +ATP/+MAPK, including its molecular weight marker (M), were run in a different gel. (PDF) [file pbio.1002184.s001.pdf]

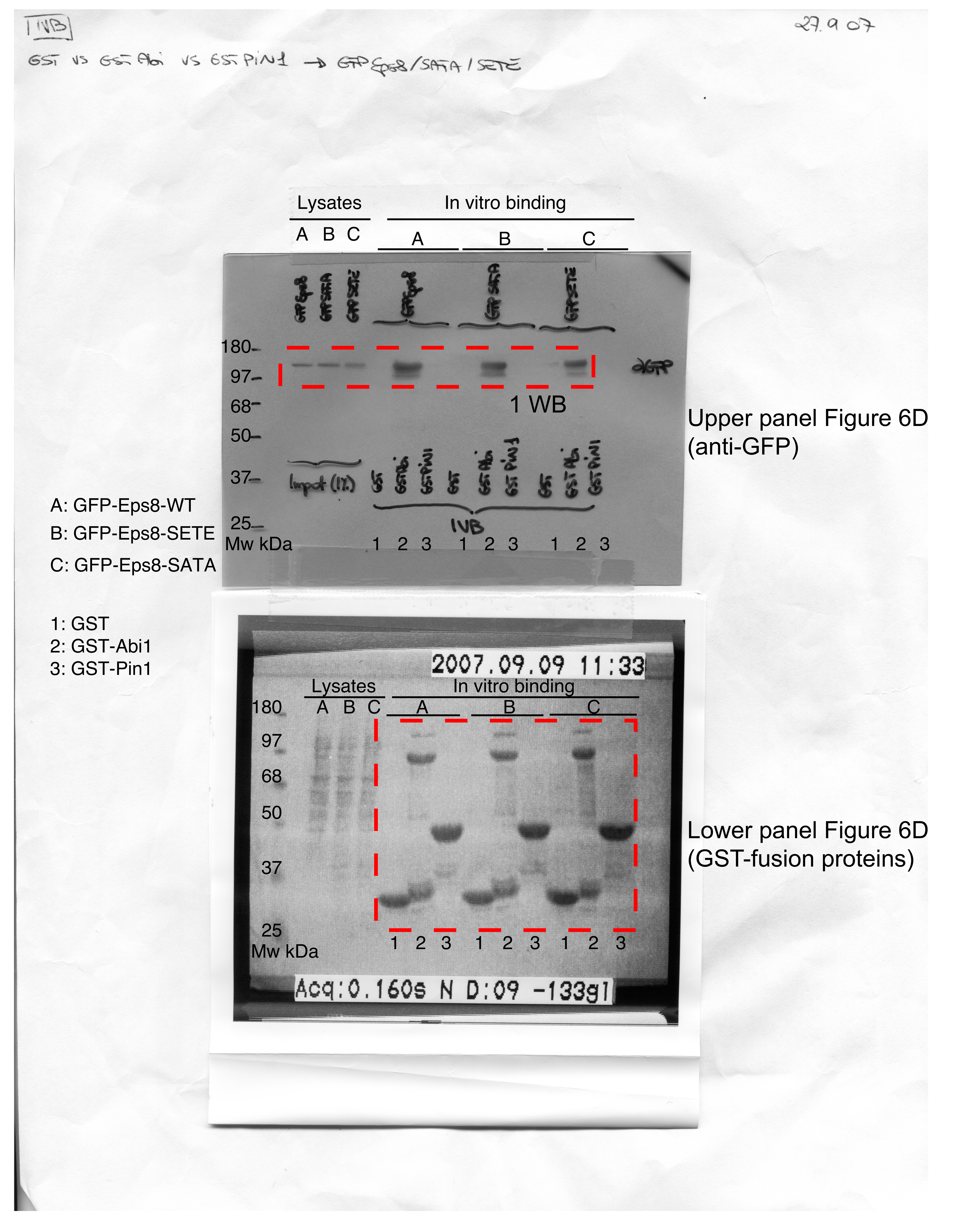

Supplement: S6 Fig — Eps8 phosphomutants bind Abi1 similar to Eps8 WT. Lysates of cells expressing GFP-Eps8 WT or SATA or SETE (indicated at the top) were incubated with immobilized GST, or GST-PIN1, used as negative control, or GST-Abi1 as described in Fig. 6D. Lysates and bound proteins were immunoblotted with anti-GFP antibody (upper panel) or stained to reveal GST (lower panels). The full scan of the original immunoblot and of the picture of the entire nitrocellulose membrane are shown. Mw markers are indicated on the left. Boxed regions indicate the cropped blot used to assemble Fig. 6D. (TIF) [file pbio.1002184.s002.tif]
